# Comparing myelin-sensitive magnetic resonance imaging measures and resulting g-ratios in healthy and multiple sclerosis brains

**DOI:** 10.1016/j.neuroimage.2022.119750

**Published:** 2022-11-13

**Authors:** Ronja C. Berg, Aurore Menegaux, Thomas Amthor, Guillaume Gilbert, Maria Mora, Sarah Schlaeger, Viola Pongratz, Markus Lauerer, Christian Sorg, Mariya Doneva, Irene Vavasour, Mark Mühlau, Christine Preibisch

**Affiliations:** a Technical University of Munich, School of Medicine, Department of Diagnostic and Interventional Neuroradiology, Munich, Germany; b Technical University of Munich, School of Medicine, Department of Neurology, Munich, Germany; c Technical University of Munich, School of Medicine, TUM Neuroimaging Center, Munich, Germany; d Philips Research Europe, Hamburg, Germany; e MR Clinical Science, Philips Healthcare, Mississauga, ON, Canada; f Technical University of Munich, School of Medicine, Department of Psychiatry, Munich, Germany; g University of British Columbia, Department of Radiology, Vancouver, BC, Canada

**Keywords:** Myelin imaging, G-ratio mapping, Myelin and axonal volume fractions, Multiple sclerosis, White matter lesions, Magnetic resonance imaging

## Abstract

The myelin concentration and the degree of myelination of nerve fibers can provide valuable information on the integrity of human brain tissue. Magnetic resonance imaging (MRI) of myelin-sensitive parameters can help to non-invasively evaluate demyelinating diseases such as multiple sclerosis (MS). Several different myelin-sensitive MRI methods have been proposed to determine measures of the degree of myelination, in particular the g-ratio. However, variability in underlying physical principles and different biological models influence measured myelin concentrations, and consequently g-ratio values. We therefore investigated similarities and differences between five different myelin-sensitive MRI measures and their effects on g-ratio mapping in the brains of both MS patients and healthy volunteers.

We compared two different estimates of the myelin water fraction (MWF) as well as the inhomogeneous magnetization transfer ratio (ihMTR), magnetization transfer saturation (MTsat), and macromolecular tissue volume (MTV) in 13 patients with MS and 14 healthy controls. In combination with diffusion-weighted imaging, we derived g-ratio parameter maps for each of the five different myelin measures.

The g-ratio values calculated from different myelin measures varied strongly, especially in MS lesions. While, compared to normal-appearing white matter, MTsat and one estimate of the MWF resulted in higher g-ratio values within lesions, ihMTR, MTV, and the second MWF estimate resulted in lower lesion g-ratio values.

As myelin-sensitive measures provide rough estimates of myelin content rather than absolute myelin concentrations, resulting g-ratio values strongly depend on the utilized myelin measure and model used for g-ratio mapping. When comparing g-ratio values, it is, thus, important to utilize the same MRI methods and models or to consider methodological differences. Particular caution is necessary in pathological tissue such as MS lesions.

## 1. Introduction

Myelin is an important constituent of neural tissue, insulating nerve fibers and enabling fast signal propagation. Measuring the distribution of myelin and the degree of myelination of nerve fibers in human brain is therefore thought to improve the evaluation and monitoring of demyelinating diseases such as multiple sclerosis (MS) (Hagiwara et al., 2017a; Laule et al., 2006).

Various myelin-sensitive magnetic resonance imaging (MRI) metrics have been developed during recent years (Mancini et al., 2020). The most established method is myelin water imaging (MWI), which measures the myelin water fraction (MWF), i.e., the quickly decaying signal arising from water trapped between myelin sheaths (Mackay et al., 1994). Other methods exploit the magnetization transfer (MT) effect between macromolecular bound and free water protons (Wolff and Balaban, 1989). MT saturation (MTsat) determines the signal decline induced by a single MT saturation pulse (Helms and Piringer, 2005), while inhomogeneous MT (ihMT) exploits the dipolar order relaxation time associated with the lipid bilayers in myelinated structures (ihMTR) (Girard et al., 2015; Varma et al., 2015) and has been suggested as being more specific to myelin than the conventional MT ratio (Duhamel et al., 2019; Ercan et al., 2018; Van Obberghen et al., 2018). Finally, the macromolecular tissue volume (MTV), a measure of the non-water volume (Mezer et al., 2013), has been used because myelin is a major constituent of non-water macromolecules in white matter (Berman et al., 2018; Norton and Autilio, 1966). Each of these contrasts has been found to correlate with myelin concentration (Berman et al., 2018; Callaghan et al., 2014; Duhamel et al., 2019; Laule et al., 2006; MacKay et al., 2006; Mezer et al., 2013) and some of them have been compared in healthy volunteers (Berg et al., 2020; Ercan et al., 2018; Vavasour et al., 2018) or in patients with MS (Berg et al., 2022; Elkady et al., 2021; Hagiwara et al., 2018; Saccenti et al., 2020).

The g-ratio, i.e., the ratio between the inner axon radius and the outer radius of the myelin sheath surrounding an axon, describes the degree of myelination of nerve fibers. Using MRI, the g-ratio can be obtained by combining myelin-sensitive and axonal-sensitive measures. The MRI-based (“aggregate”) g-ratio provides an estimate of the degree of myelination in each voxel (Stikov et al., 2015). It can be derived by combining the myelin volume fraction (MVF) obtained from myelin-sensitive measures with axonal density from diffusion MRI, e.g., obtained via neurite orientation dispersion and density imaging (NODDI) (Zhang et al., 2012). The MRI-based g-ratio is intended to provide information regarding brain white matter (WM) microstructure which is not available from other imaging parameters (Stikov et al., 2015), and it can help to disentangle ambiguities of myelin measures (a lower MVF could arise from both myelin debris and a decrease in the number of nerve cells). Various myelin-sensitive and axonal-sensitive measures have been used to calculate g-ratio values (see (Mohammadi and Callaghan, 2021) for a recent review), which have been found to correlate well with histology or electron microscopy (Stikov et al., 2015; West et al., 2018a, 2018b). However, comparing g-ratio values derived from different MRI techniques has also revealed significant differences (Campbell et al., 2018; Ellerbrock and Mohammadi, 2018).

Most clinical studies have utilized a single myelin-sensitive measure to derive the g-ratio. Only a few studies have compared different g-ratio modalities (Campbell et al., 2018; Stikov et al., 2015) in normal-appearing WM and MS lesions. However, they have mostly applied similar techniques (e.g., all based on the MT effect) and compared just two different modalities.

In order to investigate the applicability of MRI-based g-ratio mapping for clinical studies in more detail, we compared five rather different myelin-sensitive MRI parameters and derived g-ratio values in the brain tissue of 14 healthy volunteers and 13 MS patients. For calculating g-ratio values, we obtained two different estimates of the MWF as well as ihMTR, MTsat, and MTV and combined them with NODDI data. While we used established approaches from previous studies to calculate g-ratio values based on MWF, MTsat, and MTV, we suggest a novel procedure to estimate g-ratio values from ihMTR.

## 2. Methods

**Fig. 1.**
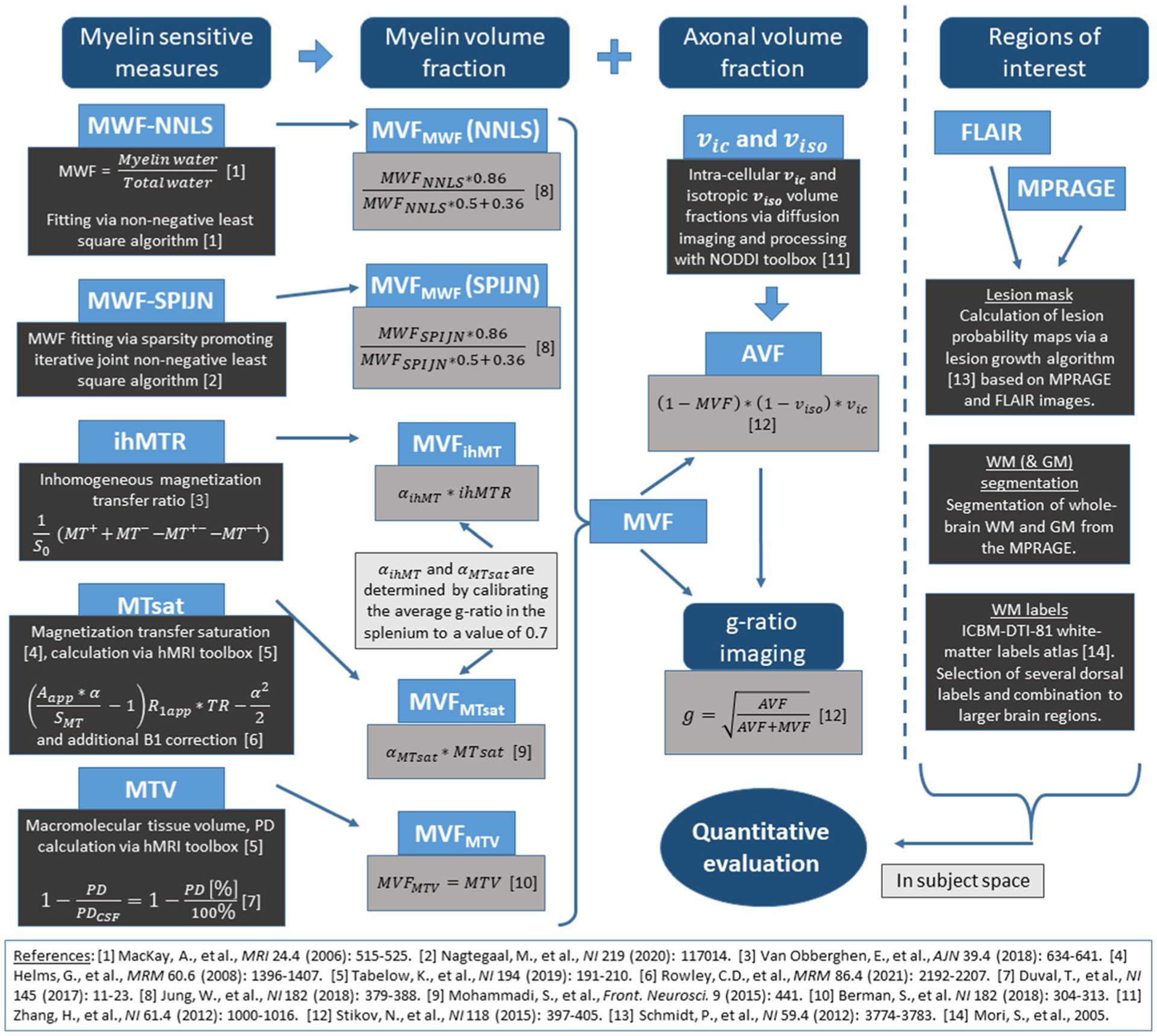
Methodological overview of acquired imaging contrasts, calculated quantitative parameters, and investigated volumes of interest. Five different myelin volume fraction (MVF) parameter values were calculated from the five different myelin-sensitive measures. Axonal volume fractions (AVF) were modeled based on diffusion-weighted MRI data and combined with MVFs to calculate g-ratio values. Quantitative evaluations were performed in MS lesions, whole brain white matter (WM) and gray matter (GM) segmentations, and several atlas-based WM regions. Please note that numbers in square brackets refer to the references provided.

Fig. 1 provides a schematic overview of the various steps of data acquisition, data processing, and parameter map calculation, which are briefly described in the following. Additional methodological details can be found in the supplementary methods.

### 2.1. Participants

This study was approved by the local medical ethics committee of the Rechts der Isar Hospital, Technical University of Munich (TUM). After providing informed written consent for participation in this study, 13 patients with MS and 14 age-matched healthy controls (HC) underwent MRI at the Department of Neuroradiology, TUM. Demographic and clinical details are provided in Table 1.

### 2.2. Image acquisition

Data acquisition was performed on a Philips Ingenia Elition X 3 T MRI system (Philips Healthcare, Best, NL; R5.6.1.0) using a 32-channel head coil. The imaging protocol consisted of magnetization prepared rapid gradient echo (MPRAGE), fluid-attenuated inversion recovery (FLAIR), 3D gradient and spin echo (GRASE) with 48 echoes for myelin water imaging (Prasloski et al., 2012b), and 3D gradient echo with three echoes and ten MT-pulses for ihMT (Girard et al., 2015). Three 3D multi-echo gradient echo data sets with T1-, PD-, and MT-weighting, including B1-mapping, were acquired for multi-parameter mapping (Tabelow et al., 2019). Diffusion-weighted imaging (DWI) data were acquired with a multiband accelerated single-shot spin echo version of an echo-planar imaging sequence with two diffusion shells (32 gradient directions at *b* = 711 s/mm^2^ and 64 gradient directions at *b* = 2000s/mm^2^ with 12 interleaved *b* = 0 s/mm^2^). In each participant, MPRAGE and FLAIR were scanned first. The order of all other sequences was permuted across participants. Detailed scanning parameters can be found in the supplementary methods and in Supplementary Table X1.

### 2.3. Data processing

If not stated otherwise, evaluations were performed using MATLAB (R2020a, The Mathworks, Natick, MA, United States). In the following, we will give a brief overview on data processing. More details are provided in the supplementary methods.

The myelin water fraction (MWF) was determined by multi-exponential fitting procedures from the 3D GRASE data using two different approaches: 1) a non-negative least squares (NNLS) algorithm (MacKay et al., 2006) including stimulated echo correction (Prasloski et al., 2012a), yielding “MWF_NNLS_”, and 2) a Sparsity Promoting Iterative Joint Non-negative least squares (SPIJN) algorithm for MWF determination (Nagtegaal et al., 2020), yielding “MWF_SPIJN_”. Inhomogeneous MT ratios (ihMTR) were calculated based on the four different 3D gradient-echo-based MT-weighted images with single (positive or negative) and dual MT saturation pulse offset frequencies according to (Girard et al., 2015). MTsat and proton density (PD) parameter maps were generated from the 3D multi-echo gradient echo data using the hMRI toolbox (Tabelow et al., 2019) included in the SPM framework (SPM12, version v7771; www.fil.ion.ucl.ac.uk/spm/software/spm12/). MTsat maps were additionally corrected for B1 inhomogeneities using a model-based approach (Rowley et al., 2021). The macromolecular tissue volume (MTV) was then obtained from the PD data, assuming all non-water protons to be macromolecules (Duval et al., 2017), and assuming PD within the cerebrospinal fluid to be 100% (Ellerbrock and Mohammadi, 2018). DWI data were preprocessed using PreQual (Cai et al., 2021), which included denoising as well as correction of susceptibility-induced distortion, motion, and eddy currents. The intracellular and isotropic signal fractions were obtained via neurite orientation dispersion and density imaging (NODDI) modeling using the NODDI toolbox (Zhang et al., 2012).

### 2.4. Calculation of myelin and axon volume fractions, and g-ratio parameter maps

g-Ratio: The g-ratio provides information on the degree of myelination of nerve fibers. Using MRI, the so-called “aggregate” g-ratio can be determined from the myelin volume fraction (MVF) and the axonal volume fraction (AVF) according to (Stikov et al., 2015)

(1) $$g=\sqrt{\frac{1}{1+\frac{MVF}{AVF}}}=\sqrt{\frac{AVF}{AVF+MVF}}.$$

In this case, the MVF was obtained from each of the acquired myelin-sensitive measures, as proposed in previous studies and described below. A number of (slightly) different scaling approaches have been proposed and are generally applied to calculate MVF from the myelin-sensitive measures. It is worth noting, however, that these procedures merely influence absolute MVF values, but will not lead to inverted contrasts.

MVF from MWF: A model of white matter tissue volumes is generally applied for calculating MVF from MWF. This model includes four compartments: myelin water volume, myelin lipid volume, non-myelin water volume, and non-myelin lipid volume. The MVF is then estimated from the MR-visible (aqueous) volume ratios of the myelin and non-myelin compartments. Given that MWF does not comprise non-water myelin components, scaling is required to calculate MVF from the MR visible myelin-associated aqueous protons. For both MWF_NNLS_ and MWF_SPIJN_, we calculated the myelin volume fraction according to (Jung et al., 2018)

(2) $$MVF_{MWF}=\frac{MWF\cdot \kappa_{nm}}{MWF\cdot \left(\kappa_{nm}-\kappa_{my}\right)+\kappa_{my}},$$

with the MR-visible volume ratios of the myelin and non-myelin compartments, *κ*_*my*_ = 0.36 and *κ*_*nm*_ = 0.86 (Jung et al., 2018), respectively.

MVF from MT: MT-based methods are intended to be sensitive to the fraction of macromolecular content, such as myelin. Since MT-based measures have been found to correlate with myelin content (Duhamel et al., 2019), a linear relationship between MVF and MTsat has been suggested (Campbell et al., 2018; York et al., 2021), which we likewise apply to ihMTR

(3) $$MVF_{MTsat}=\alpha_{MTsat}\cdot MTsat,$$

(4) $$MVF_{ihMTR}=\alpha_{ihMT}\cdot ihMTR.$$

The scaling factors *α*_*MTsat*_ and *α*_*ihMT*_ were determined by calibrating the average g-ratio in the splenium across the cohort of all healthy volunteers to a value of 0.7 (Cercignani et al., 2017; Mohammadi et al., 2015; Stikov et al., 2015).

MVF from MTV: While the macromolecular tissue volume obtained from PD (Mezer et al., 2013) is generally assumed to be linearly related to myelin content (Duval et al., 2017), previous studies have found similar values between myelin and MTV (Berman et al., 2018; Duval et al., 2017). Therefore, we felt justified in using MTV as a surrogate for MVF

(5) $$MVF_{MTV}=MTV.$$

AVF: The axonal volume fraction was calculated using the intracellular signal fraction f_ic_ and the isotropic signal fraction f_iso_ from theNODDI processing as approximate values of the respective volume fractions *ν*_*ic*_ and *ν*_*iso*_, as well as the MVF estimates according to (Stikov et al.,2015):

(6) $$AVF=(1-MVF)\cdot \left(1-v_{\text{iso }}\right)\cdot v_{ic}.$$

### 2.5. VOI definition

In all study participants (HC and MS), lesions were segmented from FLAIR and MPRAGE data using the lesion growth algorithm (Schmidt et al., 2012) from the lesion segmentation tool (https://www.applied-statistics.de/lst.html) for SPM12. Lesions were defined as segmented regions with a lesion probability > 0.5, and the resulting individual lesions were eroded by one voxel. In total, the 13 MS patients had 143 lesions remaining after erosion. Perilesional tissue (perilesion) was defined as a two-voxel-wide shell of normal-appearing white matter (NAWM) surrounding lesions. Whole-brain gray matter (GM) and WM masks were derived from lesion-filled MPRAGE data using the SPM12 “segment” module thresholded at the respective tissue probabilities > 0.9. Additionally, several anatomical regions from the ICBM-DTI-81 white-matter labels atlas (https://neurovault.org/images/1401/) (Mori et al., 2005) were combined into three WM regions (corpus callosum, corona radiata, internal capsule) and registered to the participants’ MPRAGE data using the SPM12 “normalize” module. Voxels with lesion probabilities > 0.5 were excluded from all non-lesion volumes of interest (VOIs). More information about the definition of VOI is provided in the supplementary methods.

### 2.6. Quantitative evaluations

All quantitative evaluations of parameter maps were performed in the individual subjects’ native spaces within common volumes of interest. MWF_NNLS_, MWF_SPIJN_, ihMTR, MTsat, and MTV maps were registered to the individual subjects’ MPRAGE data using trilinear interpolation. For each participant, VOI-mean values of myelin-sensitive measures, volume fraction maps, and g-ratios were extracted from whole-brain WM segmentations and atlas-based WM regions. For MS patients, evaluations were additionally performed in lesion and perilesion tissue. The MVF and AVF values were correlated with each other in WM of healthy volunteers, as well as in NAWM and in WM lesions of MS patients. Finally, correlations between VOI-mean g-ratio values calculated from the five different myelin-sensitive measures were performed for all ten combinations of g-ratio pairs within healthy WM, NAWM, and lesion segmentations.

Two-sample *t*-tests were performed using the MATLAB “ttest2” function to assess the statistical significance of differences between healthy WM and NAWM, and between NAWM and (peri-) lesion tissue. These tests were performed for each of the myelin-sensitive measures and each of the five g-ratio measures. Additionally, Pearson correlation coefficients were calculated between pairs of subject-mean values from different myelin-sensitive measures and different g-ratio values to evaluate their correlation within healthy WM, NAWM, and MS lesions. For more details, see the supplementary methods.

### 2.7. Data and code availability statement

In line with local ethics guidelines and participant privacy policies, the sharing of acquired data will be considered upon reasonable request. Institutional policies would then require a formal data sharing agreement.

For NNLS-based MWF calculation, the MATLAB scripts can be obtained from https://mriresearch.med.ubc.ca/news-projects/myelin-water-fraction/ and the Decaes toolbox provides a Julia-based equivalent for data processing (https://github.com/jondeuce/DECAES.jl). The processing script for ihMTR calculation can be made available upon request. The software for SPIJN-based MWF calculation requires a formal research agreement with Philips. A demo version is available via https://github.com/MNagtegaal/SPIJN. The latest version of the hMRI toolbox, which was used for calculation of MTsat and PD parameter maps, is available from www.hMRI.info. The latest version of the NODDI toolbox can be downloaded from https://www.nitrc.org/projects/noddi_toolbox. Sharing of any sequence modification applied here is limited by a nondisclosure agreement with the scanner manufacturer.

## 3. Results

### 3.1. Myelin-sensitive measures

**Fig. 2.**
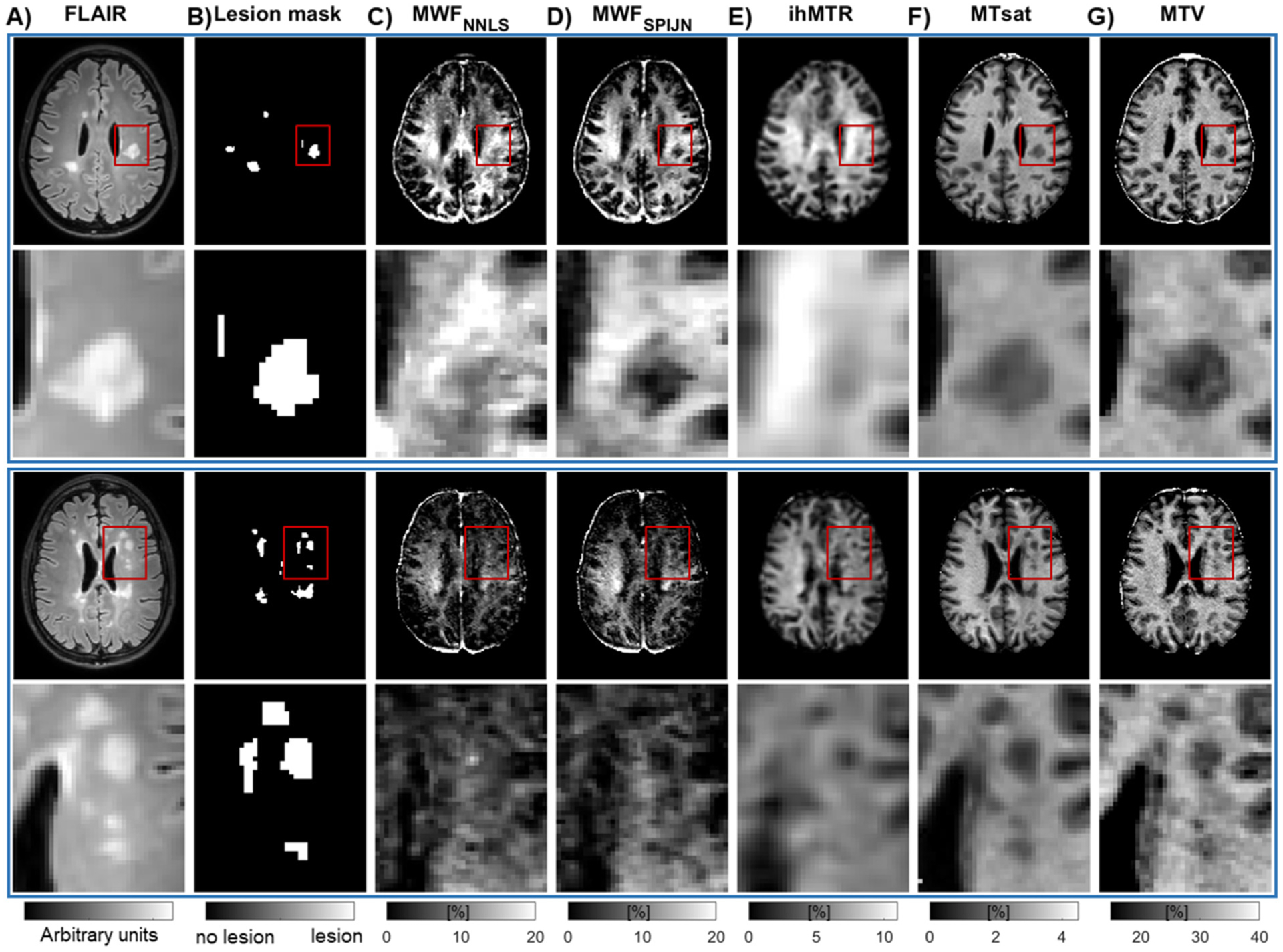
Representative slices of FLAIR (A), lesion segmentations (B), and maps of myelin-sensitive markers (C-G) from two MS patients, including expanded regions. Myelin-sensitive markers include two reconstructions of the myelin water fraction, MWF_NNLS_ (C) and MWF_SPIJN_ (D), inhomogeneous magnetization transfer ratio (ihMTR) (E), MT saturation (MTsat) (F), and macromolecular tissue volume (MTV) (G). Red rectangles in the first and third rows indicate the location of the zoomed-in regions in the second and last rows.

Overall, the parameter maps of the acquired myelin-sensitive measures were similar in appearance and generally showed higher parameter values in WM areas than in GM (Fig. 2). However, the parameter maps varied in the degree of smoothness and contrast between different brain regions, especially in lesions. While some lesions appeared dark in all contrasts, others varied strongly in the degree of parameter decrease compared to surrounding NAWM (Fig. 2).

**Fig. 3.**
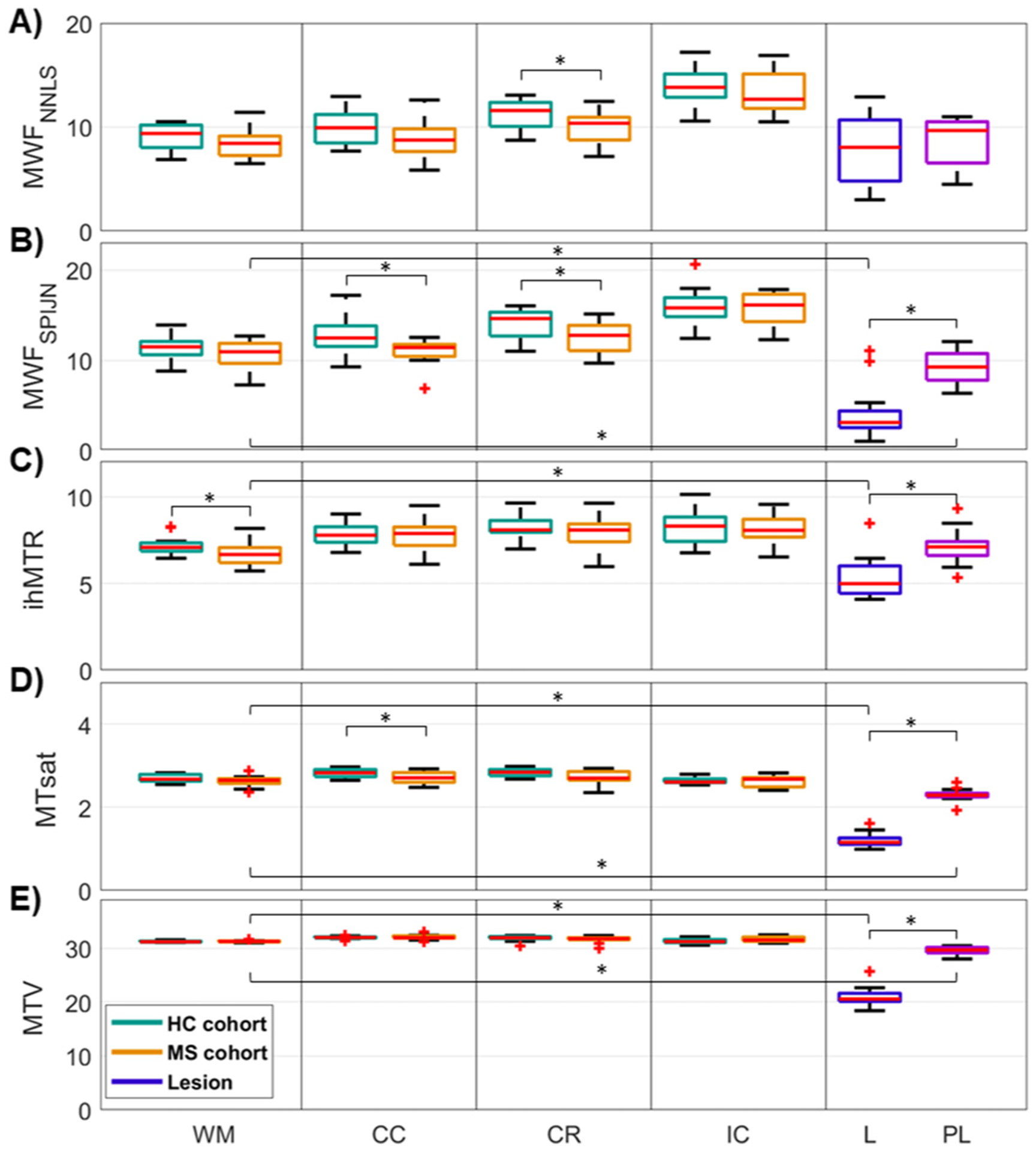
Boxplots of subject-mean myelin-sensitive measure values within several different VOIs. The values of MWF_NNLS_, MWF_SPIJN_, ihMTR, MTsat, and MTV were evaluated for healthy (HC cohort, green) and normal-appearing (MS cohort, orange) white matter regions, in MS lesions (purple-blue), and in a 2-voxel wide shell around MS lesions (“perilesion”, pink). In all five panels, the boxplots represent distributions across subjects. Significant differences between HC and MS cohorts within the same VOI or between MS WM, lesion, and perilesion are indicated by asterisks. Abbreviations: WM: whole-brain WM, CC: corpus callosum, CR: corona radiata, IC: internal capsule, L: lesion, PL: perilesion.

**Fig. 4.**
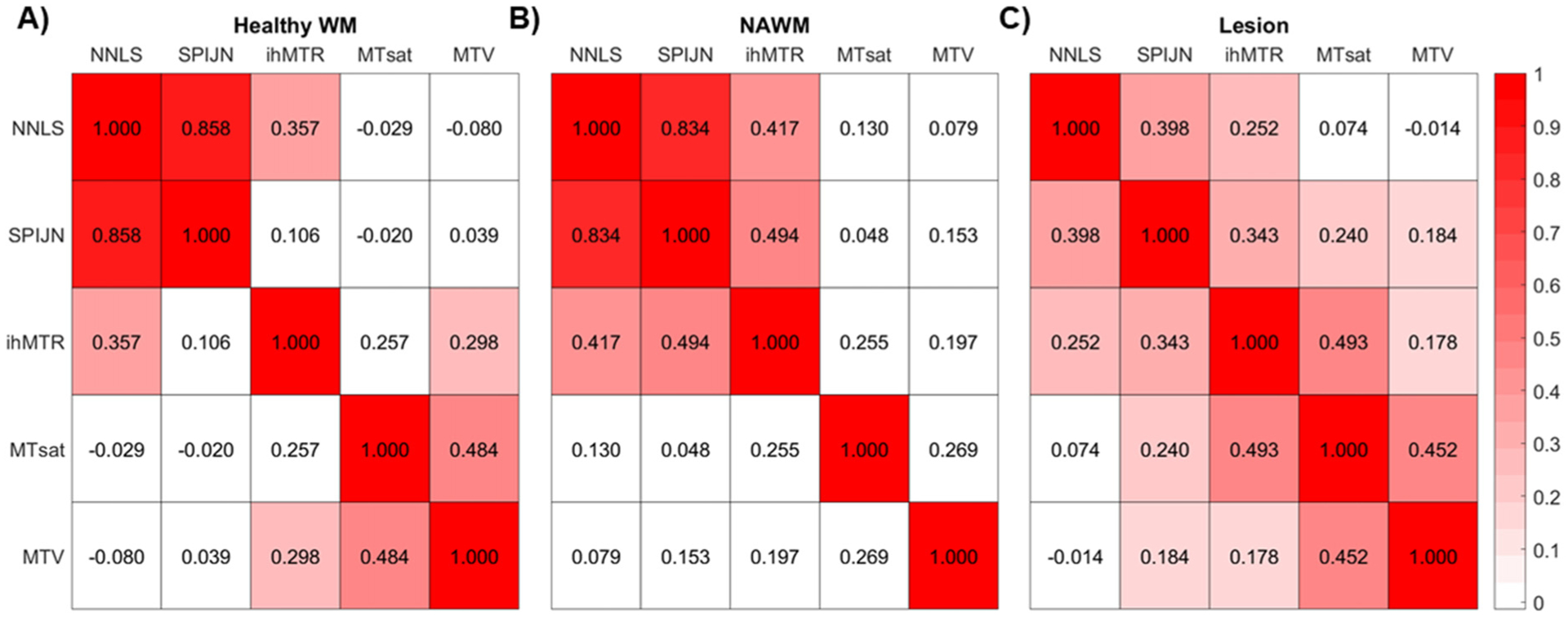
Pearson’s correlation coefficients between all combinations of subject-mean values from different myelin-sensitive measures within several brain regions. Heat maps of correlation coefficients are illustrated as 2D symmetric matrices and shown for healthy WM (A), as well as NAWM (B), and lesions (C) of MS patients. Non-significant correlations between myelin-sensitive markers (*p*-values > 0.05) are shown in white. WM: whole-brain white matter, NAWM: normal-appearing white matter, NNLS: MWF_NNLS_, SPIJN: MWF_SPIJN_.

Quantitative evaluations of myelin-sensitive parameters revealed larger differences between lesion and whole-brain NAWM for MWF_SPIJN_, MTsat, and MTV and smaller differences for ihMTR (Fig. 3). For MWF_NNLS_, no statistically significant difference was found between lesion and NAWM but for both MWF-based contrasts, healthy WM and NAWM differed significantly in some of the WM VOIs (Supplementary Table X2). Differences between various WM VOIs were most prominent for MWF_NNLS_ (Fig. 3 and Supplementary Figure S3 for additional VOIs). Several myelin-sensitive measures were found to correlate significantly with each other in WM (Fig. 4). In lesions, all combinations of myelin-sensitive measures showed significant correlations, except for MWF_NNLS_ with both MTsat and MTV (Fig. 4).

### 3.2. Comparisons of MVF, AVF, and g-ratio maps

**Fig. 5.**
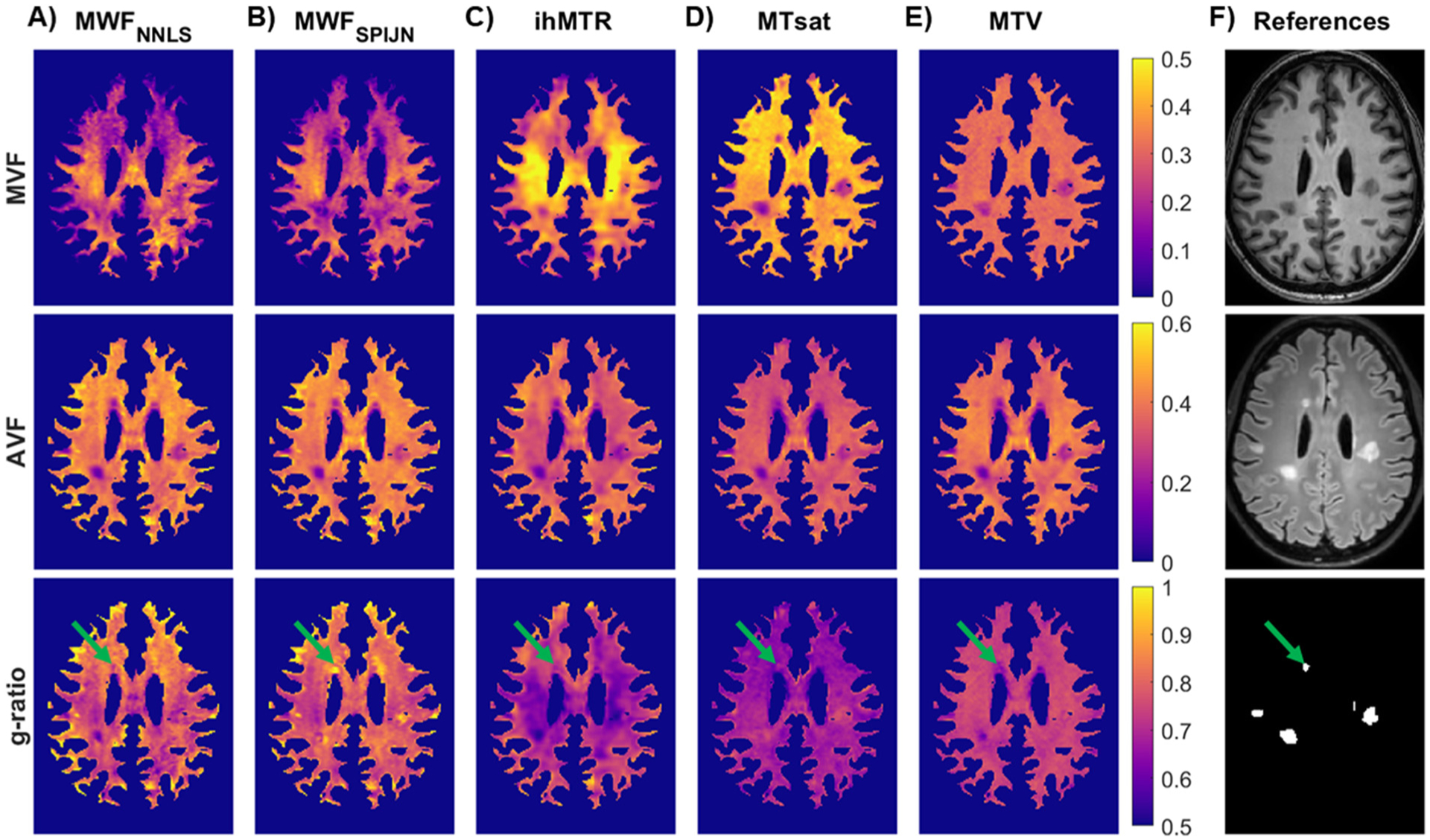
Representative slice of myelin volume fraction (MVF), axonal volume fraction (AVF), and g-ratio parameter maps from an MS patient calculated based on different myelin-sensitive measures. Myelin-sensitive measures include MWI using two different fitting techniques for the myelin water fraction, MWF_NNLS_ (A) and MWF_SPIJN_ (B), as well as ihMTR (C), MTsat (D), and MTV (E). As reference, the MPRAGE (top), FLAIR (middle), and lesion mask (bottom) are shown in the last column (F). Green arrows point to an MS lesion that shows differences in the g-ratio values between different g-ratio mapping methods.

The visual appearance of MVF, AVF, and g-ratio maps differed somewhat, depending on the myelin-sensitive measure from which they were calculated (Fig. 5 and Supplementary Figure S4). MWF-based measures resulted in the lowest MVF and highest g-ratio values while ihMTR, MTsat, and MTV provided slightly higher MVF but lower g-ratio values. Especially in lesions, g-ratio values based on different myelin-sensitive measures revealed diverging behaviors, with (compared to NAWM) higher g-ratio values in some parameter maps and lower values in others (Fig. 5, green arrows).

**Fig. 6.**
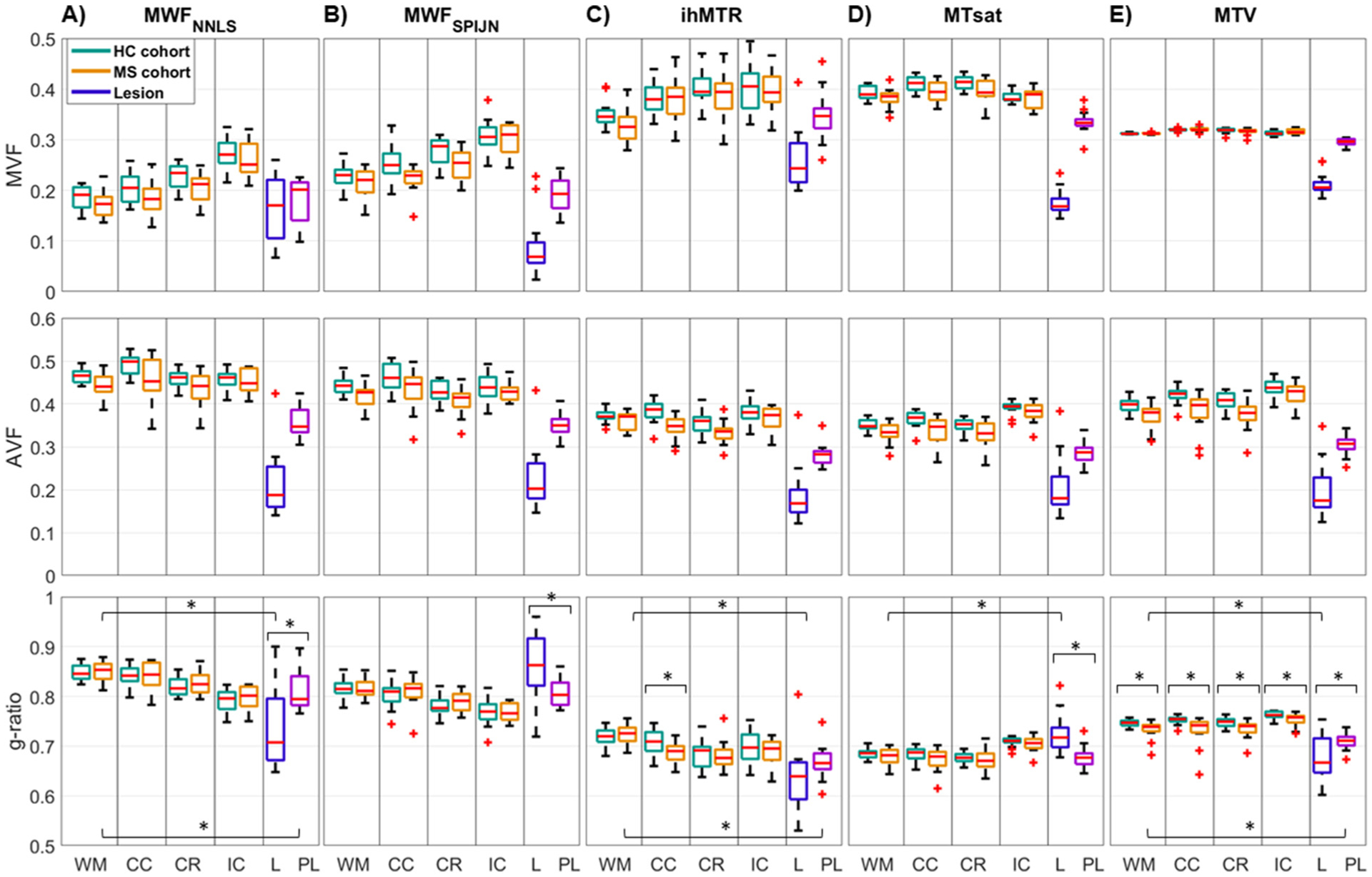
Quantitative comparison of subject-mean MVF, AVF, and g-ratio values in several brain regions. The volume fractions were calculated for each of the five myelin-sensitive measures (columns) and evaluated in healthy (HC cohort) or normal-appearing (MS cohort) white matter regions, in MS lesions, and in a 2-voxel wide shell around MS lesions (“perilesion”). For each of the five g-ratio measures, statistically significant differences between healthy WM and NAWM or between whole-brain NAWM and (peri-) lesion tissue are indicated by an asterisk. Abbreviations: WM: whole-brain WM, CC: corpus callosum, CR: corona radiata, IC: internal capsule, L: lesion, PL: perilesion.

VOI-mean intracellular volume fractions were strongly decreased in lesion and perilesion compared to WM, while VOI-mean isotropic volume fractions obtained from NODDI modeling varied only slightly across white matter regions, as well as within lesion or perilesion (Supplementary Figure S5). Regarding MVF, VOI-mean values showed quite some variability across imaging methods, especially within lesions compared to NAWM (Fig. 6, top row). Considering outliers, similar variances across subject-mean lesion values were found for MWF_NNLS_, MWF_SPIJN_, and ihMTR ranging from clearly reduced g-ratio values to values comparable to NAWM. MTsat and MTV showed a much smaller variance of subject-mean values both in lesion segmentations and in WM VOIs (Fig. 6, top row). Derived VOI-mean AVF values, on the other hand, were actually quite comparable (Fig. 6, middle row). The largest variability was found when comparing g-ratio values within lesions. While average g-ratios within lesions were lower than within healthy WM and NAWM for MWF_NNLS_, ihMTR, and MTV, they were higher when calculated based on MWF_SPIJN_ and MTsat (Fig. 6, bottom row). Similar results were obtained when comparing MVF, AVF, and g-ratio values in additional WM VOIs and in the lesion shell, i.e., the outermost 1-voxel wide layer segmented as a lesion, which can provide information about the lesion homogeneity within the segmentation when compared to the lesion center (Supplementary Figure S6). The g-ratio values within lesions differed significantly from whole-brain NAWM for all methods except for g-ratio values calculated from MWF_SPIJN_ (Supplementary Table X3). For most investigated WM VOIs, g-ratio values did not differ significantly between healthy WM and NAWM (Fig. 6 and Supplementary Table X3).

### 3.3. Correlations of MVF, AVF, and g-ratio values

**Fig. 7.**
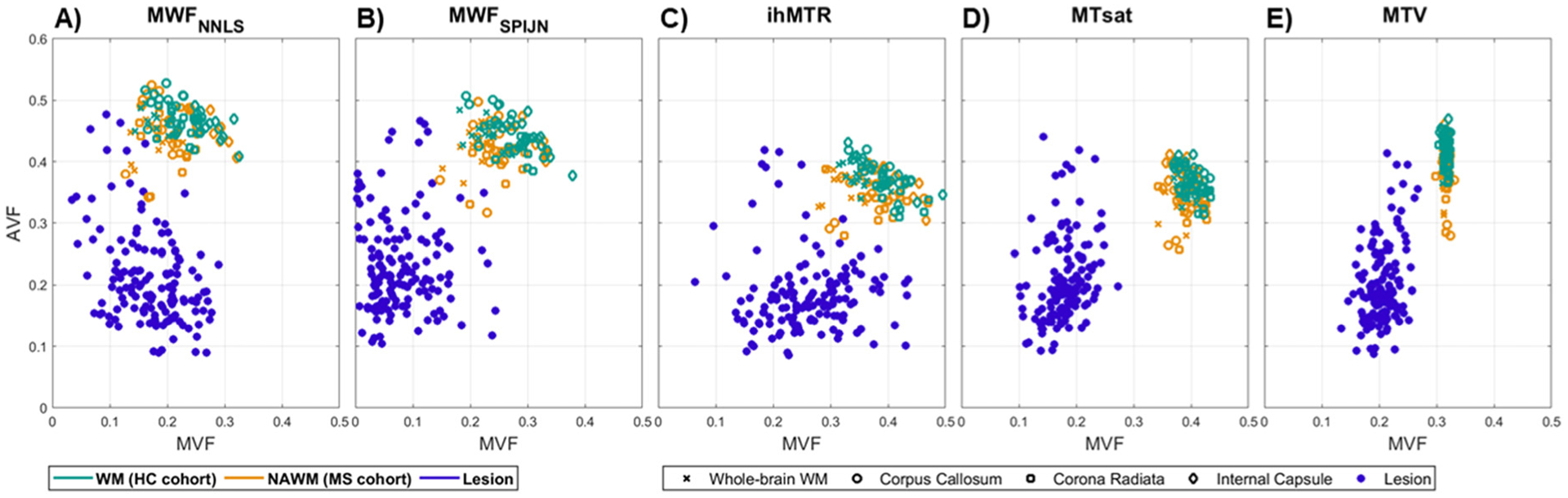
Scatterplots of MVF and AVF values calculated from different myelin-sensitive contrasts and evaluated in several WM regions and in individual lesions. The individual VOI-mean AVF values calculated from MWF_NNLS_ (A), MWF_SPIJN_ (B), ihMTR (C), MTsat (D), and MTV (E) are plotted against respective MVF values. The evaluations were performed in whole-brain WM and three atlas-based WM regions of healthy volunteers (green) and MS patients (orange), and in each individual segmented MS lesions of all 13 patients (purple-blue).

Scatterplots between VOI-mean MVF and AVF values revealed a similar range of AVF within individual lesions for data acquired with different myelin-sensitive measures but demonstrated differences in the range of MVF values (Fig. 7, purple dots). While ihMTR resulted in the largest range of MVF values within lesions (0.06–0.44), MTsat- and especially MTV-based MVF exhibited a much smaller range (0.09–0.27 and 0.13–0.27, respectively) within lesion segmentations (Fig. 7).

**Fig. 8.**
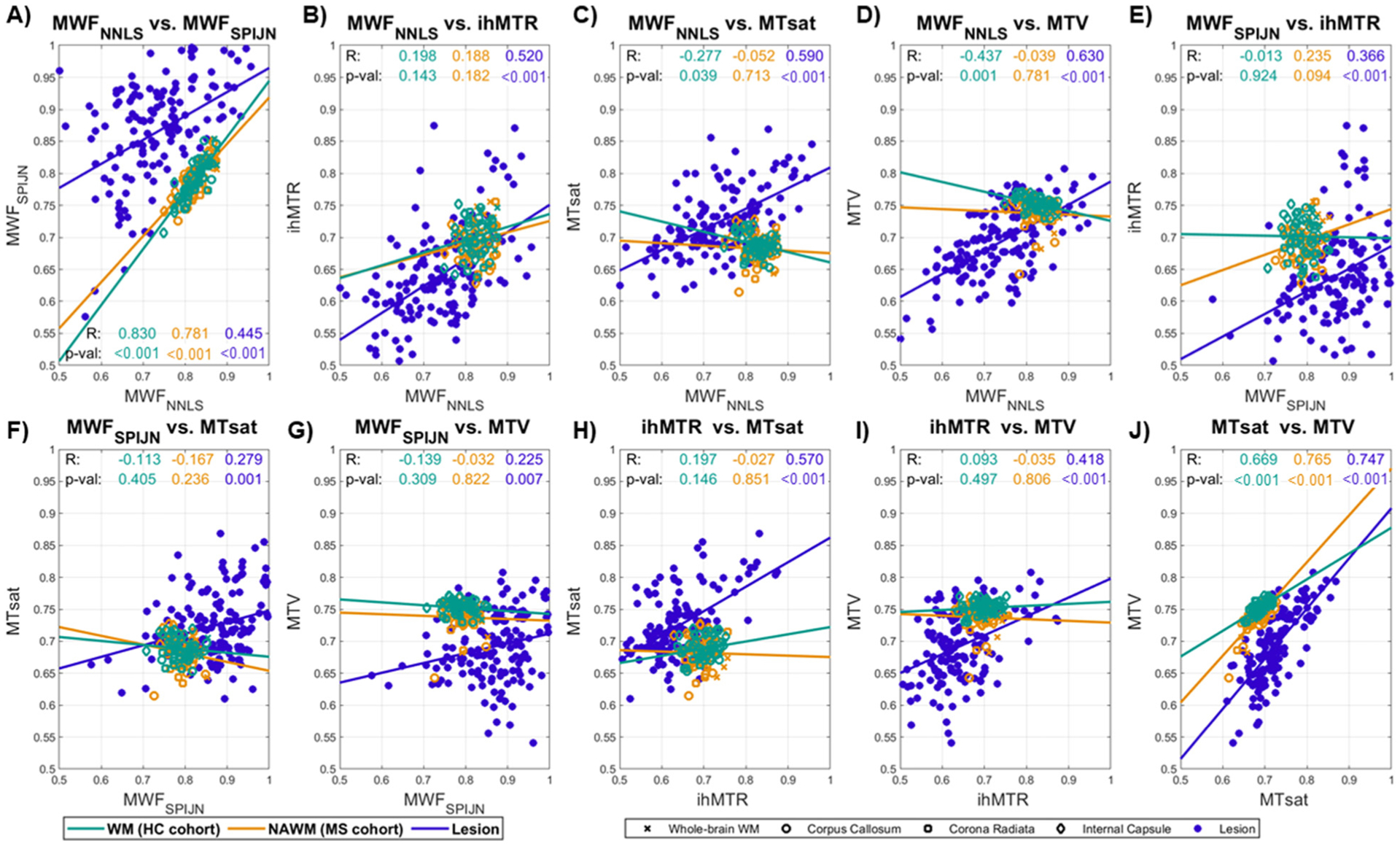
Correlations between VOI-mean g-ratio values calculated from different myelin-sensitive measures. The plots A) through J) show correlations of the five acquired myelin-sensitive measures in healthy (green) and normal-appearing (orange) white matter regions, and in lesions (purple-blue) for each combination of measures. Data points from different VOIs are represented by different marker types and colors. The Pearson correlation coefficient (R) and *p*-value (p-val) are also listed for each tissue type.

The g-ratio values calculated from both MWF-based measures correlated well in both healthy WM and NAWM. Furthermore, g-ratios based on MTsat and MTV correlated significantly in all tissue types (Fig. 8). Other pairs of g-ratio values within WM regions demonstrated only much lower and mostly non-significant correlations. Within lesions, however, all ten combinations of g-ratio values correlated significantly (Fig. 8).

## 4. Discussion

In this study, we compared five different myelin-sensitive measures in healthy and multiple sclerosis brains, namely two different estimates of the MWF (MWF_NNLS_ and MWF_SPIJN_), ihMTR, MTsat, and MTV, in order to investigate similarities and differences, and their effects on g-ratio imaging. The g-ratio parameter maps were calculated by combining myelin-sensitive parameters with intracellular and isotropic volume fractions from NODDI evaluations. The highest correlations were found between the two MWF-based myelin measures, between both MWF-based measures and ihMTR, and between MTsat and MTV. Overall, the MVF, AVF, and g-ratio maps calculated from different myelin measures appeared somewhat similar, but varied in intensity. Quantitative evaluations revealed strongest differences between the five g-ratio measures within segmented lesions. Most strikingly, MWF_SPIJN_ and MTsat exhibited increased g-ratio values in lesions compared to whole-brain WM, while MWF_NNLS_, ihMTR, and MTV showed decreased g-ratios. These results emphasize that both myelin-sensitive measure and data processing can have a crucial impact on resulting g-ratio values, especially in pathological tissue such as MS lesions.

### 4.1. Myelin-sensitive measures

Both MWF_NNLS_ and MWF_SPIJN_ in whole-brain healthy WM and NAWM (8.4–11.3% in healthy WM and 8.3–10.6% in NAWM) agreed with previous studies (Oh et al., 2007; Tozer et al., 2005), but were generally at the lower end of MWF values in the literature (Alonso‐Ortiz et al., 2015). In this context, possible reasons may be a lower signal-to-noise (Wiggermann et al., 2020) for our protocol using an echo spacing of 8 ms and scanner-specific hardware differences, which can affect MWF values (unpublished data, Irene Vavasour). In lesions, the average MWF_NNLS_ was 8.0%, which was within the range of literature values (4.6–8.0%) (Laule et al., 2004; Oh et al., 2007; Tozer et al., 2005), whereas MWF_SPIJN_ was consistently lower (4.1%). However, variably decreased MWF has been found in lesions (Laule and Moore, 2018), ranging from 0 to 17% (Laule et al., 2004), which covers our MWF_SPIJN_ results.

MT-based measures can be influenced by the properties of the magnetization transfer RF pulse (Teixeira et al., 2019), which complicates comparison with the literature. For ihMTR, this effect is smaller, and our measured values were well within the range of the literature (5.4–8.3% in healthy WM, 4.4–8.0% in NAWM, and 3.6–7.2% in lesions) (Rasoanandrianina et al., 2020; Van Obberghen et al., 2018). For MTsat, the parameter dependence is usually stronger, but relative differences between tissue types can be compared. In our study, MTsat values in lesions were ~50% lower than in NAWM, which was comparable to the literature (42% difference between lesions and NAWM) (Saccenti et al., 2020).

Finally, our MTV values were slightly higher than those previously observed (27% in healthy WM, 25% in NAWM, and 17% in lesion) (Yu et al., 2019), but the fractional difference between MTV in NAWM and lesion segmentations was comparable. Generally, our MTV values exhibited a low level of variability within WM VOIs across participants, which was likely caused by the calibration of the underlying PD values to 69% in whole-brain WM.

The myelin-sensitive measures investigated relied on different physical principles. While MWF measures the water trapped between myelin sheaths, ihMTR, MTsat, and MTV are rather based on macromolecular content, however, with a higher specificity of ihMTR to myelin compared to MTsat and MTV. Accordingly, we found significant correlations between both MWF-based measures in WM as well as in lesions. We also found significant correlations between MWF_NNLS_ and ihMTR, and between MWF_SPIJN_ and ihMTR (Fig. 4). These findings fit with the higher specificity of both MWF and ihMTR to myelin. On the other hand, good correlations were found between MTsat and MTV, which were most likely due to their common sensitivity to macromolecular content.

As expected, all myelin-sensitive measures exhibited roughly a similar behavior within white matter VOIs, with slightly lower values in whole-brain WM and slightly higher values in the internal capsule and the splenium. However, we observed clear differences within the lesion VOIs, which are most likely also affected by differences in the sensitivity and specificity of the myelin-sensitive measures to both myelin and non-myelin macromolecules. Interestingly, MWF_NNLS_ showed no significant difference between lesions and NAWM, whereas all other myelin-sensitive measures did. This finding is specifically striking for MWF_SPIJN_, which was derived from the very same data as MWF_NNLS_. While MWF_SPIJN_ was comparable although slightly higher in WM VOIs compared to MWF_NNLS_, it was clearly lower in lesions (Fig. 3). The observed differences undoubtedly result from the different technical implementations of the two processing methods. Both methods try to solve the highly ill-conditioned problem of calculating the MWF using some kind of regularization. Differences in the regularization can lead to different results, because using no regularization leads to large errors (due to noise amplification and error propagation) and too much regularization increases bias caused by the assumptions made in the regularization. In the SPIJN algorithm, a global regularization is utilized, i.e., the joint sparsity constraint, which restricts the T2 distribution to a small set of relaxation times shared between all voxels (Nagtegaal et al., 2020), whereas in NNLS voxel-wise fitting and an L2-norm regularization are utilized to achieve a smooth T2 distribution (Wiggermann et al., 2020). Regions with low MWF such as lesions are likely to suffer more from noise in the measurements. This could lead to varying T2 values and noisier images in NNLS, while SPIJN produces a more consistent spatial MWF distribution due to the global restriction to fixed T2 values. Furthermore, both methods may suffer from inaccuracies in the fitting procedures that specifically emerge in pathological tissue. The divergence of MWF values in MS lesions demonstrates that at least one of the two algorithms failed to provide reliable MWF values in MS pathology, so comparisons with histology in pathological tissue samples are urgently needed to evaluate which method provides the more realistic myelin estimate. In our opinion, the similar results from both MWF fitting algorithms in healthy tissue results from the fact that both methods were developed and optimized using healthy volunteer data. We thus conclude that it is insufficient to develop and optimize quantitative MRI techniques on data from healthy volunteers as this might lead to failure in the case of pathology.

We also found significant differences between perilesion and NAWM tissue using MWF_SPIJN_, MTsat, and MTV, which was in line with the results from previous studies (Hagiwara et al., 2017b; Saccenti et al., 2020). Differences between healthy WM and NAWM were found only for some combinations of WM VOIs and myelin-sensitive measures, but most of the VOIs and myelin measures exhibited no significant differences (Fig. 3). This outcome was in line with a previous study (Tozer et al., 2005), but several other groups observed a clear reduction in myelin measures between WM of MS patients and healthy controls (Faizy et al., 2016; Oh et al., 2007; Van Obberghen et al., 2018; Yu et al., 2019). This was most likely caused by the comparatively low EDSS and the short disease duration of our small cohort of MS patients. Furthermore, MWF reductions in NAWM have been found to vary with the subtype of MS, and greater myelin loss has been found in the more progressive forms (Kitzler et al., 2012), whereas most of our study participants suffered from relapsing-remitting MS.

For neuroscientific applications, the combination of several complementary myelin-sensitive measures could help in better understanding tissue integrity and pathological changes (Lazari and Lipp, 2021). In this respect, MWF maps showing larger differences between several WM VOIs and being expected to have the highest specificity for myelin could be combined with MTsat, which enables a clear differentiation between lesion, perilesion, and NAWM at a high spatial resolution. In this context, MWF_SPIJN_ in particular could be a suitable option, i.e., revealing significant differences between healthy WM and NAWM in several WM VOIs and, similar to MTsat, between lesion, perilesion, and NAWM. In addition, these two methods could be more feasible in clinical routines than ihMTR or MTV. In contrast to MWF and MTsat, ihMTR has the drawback of requiring pulse sequence programming, and it has a rather low spatial resolution, while MTV reveals rather small differences between WM regions or between healthy WM and NAWM, possibly caused by a lower specificity to myelin and the calibration of the underlying PD values.

### 4.2. NODDI-based intracellular volume fractions

The intracellular volume fractions in healthy WM, NAWM, and within the lesion segmentations obtained in this study (Supplementary Figure S5) agreed well with those of some previous studies (Andersen et al., 2018; Hagiwara et al., 2019). However, reference *ν_ic_* values varied strongly between different previous studies, possibly affected by the number of shells and absolute b-values as well as echo and repetition times used for diffusion imaging (Supplementary Table X4). Common across all studies were the clearly reduced *ν_ic_* values within lesions compared to WM, which was in good agreement with our findings.

### 4.3. MVF and AVF values

The calculated MVF and AVF values of most myelin-sensitive measures in WM agreed with those from two previous studies, which obtained average MVF values of 0.27 and 0.31 and average AVF values of 0.24 and 0.44 (Hagiwara et al., 2017a; Yu et al., 2019), respectively. Solely MWF-based methods resulted in slightly lower MVF as well as higher AVF (Fig. 6). Possible explanations could be that the other methods (including those utilized in the literature) slightly overestimated MVF or that the MWF-based methods slightly underestimated MVF. The latter effect would be in line with previous findings that MWF-based myelin volume fractions slightly underestimated histological MVF (West et al., 2018b). In our study, AVF lesion values were about 50% lower compared to NAWM and, for most myelin measures, the axonal decline appeared larger than the decline of myelin volume. In histological studies, transection of axons has been found to play a major role in MS and to be related to the degree of inflammation within the lesion (Trapp et al., 1998). In histological examinations of postmortem brains of mainly progressive forms of MS, a 49% decline of axonal content was found in lesions (Laule et al., 2013), and in high EDSS spinal cord lesions, axonal loss was found to range from 45% to 84% and averaged 68% (Bjartmar et al., 2000). Despite being measured in high grade MS post mortem samples, these results indicate a potentially high influence of MS on axonal volume, which would be in line with the findings in this study.

### 4.4. White matter g-ratio values

The g-ratio values within healthy WM and NAWM calculated from different myelin-sensitive measures varied between 0.68 (MTsat) and 0.85 (MWF_NNLS_), where differences resulted from the myelin measure used. These findings agree with several previous studies, which also found differences between g-ratio values measured using different myelin- or axonal-sensitive measures (Campbell et al., 2018; Ellerbrock and Mohammadi, 2018; West et al., 2018b). The ihMTR-, MTsat-, and MTV-based g-ratio values roughly conformed to the literature, whereas g-ratios calculated from both MWF methods were slightly increased (Table 2). This finding may have been influenced by the ten-dency of MWF-based MVF to slightly underestimate histological MVF (West et al., 2018b) and the fact that our MWF values were at the lower end of values found in previous studies.

In this study, we used the most established approaches for g-ratio imaging. However, given that these are based on different models and model parameters, they resulted in somewhat different g-ratio values. A comparison of MWF-based g-ratio values using several different tissue models (Supplementary Figure S1) revealed slightly increased values compared to the literature (Table 2) for all of the models investigated. Likewise, the MTsat values varied depending on the acquisition parameters and MT-based g-ratio imaging thereby requires some form of calibration. While the splenium is most commonly used as calibration region, its g-ratio value is still subject to debate and affects the resulting g-ratio values (Supplementary Figure S2).

### 4.5. Lesion g-ratio values

In accordance with our findings, a high variability of MRI-measured g-ratio values was found within lesions, ranging from 0.65 (Campbell et al., 2018) to 0.93 (Hagiwara et al., 2017a). Generally, g-ratio values are expected to be higher within MS lesions than in NAWM due to a loss of myelin bilayers, where older lesions demonstrate the greatest myelin loss (Laule and Moore, 2018). This finding was confirmed in a number of studies using MRI for g-ratio mapping (Hagiwara et al., 2017a; Maekawa et al., 2022; Yu et al., 2019) (Table 2). However, one previous study found an unrealistically low g-ratio value within lesions compared to NAWM (Campbell et al., 2018), possibly caused by incorrect model assumptions such as a non-linear relationship between MT ratio and MVF or T1 contamination in the MT ratio map compared to the T1-corrected MTsat map. Theoretically, a decreased g-ratio value within a lesion compared to NAWM is possible if mostly thinly myelinated axons are lost or axons are transected. Overall, our findings (Fig. 6) agreed well with previous results (Table 2).

While MVF values obtained from different myelin-sensitive measures were generally lower in lesion than in NAWM VOIs, they varied in absolute numbers. MVF in lesions was lowest for MWF_SPIJN_ (MVF: 0.09), slightly higher for MWF_NNLS_ (MVF: 0.17) and MTsat (MVF: 0.18), and highest for MTV (MVF: 0.21) and ihMTR (MVF: 0.26). Since the AVF values were comparable between methods, these differences in absolute MVF values had a strong influence on the resulting g-ratio values. While MWF_SPIJN_ and MTsat showed, on average, a higher g-ratio within lesions than within WM, MWF_NNLS_, ihMTR, and MTV indicated lower g-ratio values compared to WM VOIs (Fig. 6). These findings show that at least two of the investigated myelin-sensitive measures failed to provide accurate information on g-ratio values and pathological alterations of the degree of myelination in MS lesions. Most striking were the differences between ihMTR and MTsat, which both rely on the magnetization transfer effect, and even more between MWF_SPIJN_ and MWF_NNLS_, which were derived from identical data. Differences between both MT-based g-ratio values were certainly influenced by the higher sensitivity and specificity of ihMTR to myelin compared to MTsat. Further influencing factors (in arbitrary order) may have been white matter fiber orientation-dependent differences of both MT contrasts (see Section 4.7), unaccounted B1 field influences in the ihMTR maps, a lower signal-to-noise due to the smaller ihMT effect compared to regular MT, a non-linear relation between ihMTR and MVF, or a single-point calibration insufficiency (Mohammadi and Callaghan, 2021). Additionally, the sensitivity and specificity of ihMTR are dependent on sequence parameters (Duhamel et al., 2019) and may thus depend on our choice of imaging parameters. Differences between both MWF-based methods could have emerged from different regularization and fitting procedures, as described in Section 4.1.

This strong dependency of measured g-ratios on the myelin-sensitive MRI markers in (normal-appearing) white matter and particularly in lesions, as well as on model parameters to obtain MVF and AVF highlights that caution needs to be exercised when considering MRI-based measures of aggregate g-ratio. In line with previous studies (Campbell et al., 2018; Ellerbrock and Mohammadi, 2018), our study demonstrates the importance of considering methodological differences when comparing g-ratio values based on different MRI methods, as well as the need for more standardization. In the current situation, we agree with (Campbell et al., 2018), who proposed using the term “g-ratio-weighted” imaging. In the future, more work is clearly needed to validate MRI-based g-ratio values obtained from different myelin-sensitive measures with gold standard techniques, e.g., comparison to histological examinations.

### 4.6. Limitations of the g-ratio model in pathology

Our results demonstrated a considerable degree of variability in the measured g-ratio values within lesions, which indicates validity issues with the g-ratio model. According to the design of our study, the variability across methods strongly depends on the employed myelin-sensitive measures. More specifically, several assumptions of the g-ratio model could potentially be invalid and thus lead to inaccuracies in pathological tissue. To name but a few, compositional differences within lesions compared to normal-appearing or healthy WM could lead to a fitting bias (e.g., in MWF mapping). Likewise, an increased number of non-myelin molecules within lesions could lead to overestimated MTsat or MTV values, and finally, the relation between myelin-sensitive measures and MVF could not be linear in the case of pathology.

Furthermore, inaccuracies in the AVF estimation using NODDI can also be expected to influence the accuracy and specificity of the g-ratio estimates, especially in pathological tissue. First, general limitations (not specific to pathology) of NODDI are that it uses fixed model parameters for the intrinsic free diffusivity and perpendicular extracellular diffusivity and that it does not include complex fiber distributions such as fanning or crossing fibers in the model (Zhang et al., 2012). With respect to the latter limitation, more advanced techniques such as b-tensor encoding (Cottaar et al., 2020) could be used to improve the fiber dispersion estimation, and thereby the accuracy of the AVF estimates.

Second, NODDI-derived signal fractions have been found to depend on compartment-specific T2 relaxation times, and multi echo time diffusion imaging has been suggested to remove T2-dependencies in the signal fractions (Gong et al., 2020). Alternatively, the obtained signal fractions could be corrected for the T2 relaxation times of different tissue compartments (Papazoglou et al., 2022) if the T2 times of each compartment (isotropic, intra-, and extracellular) were known. However, for lesions (and even healthy tissue), the T2 relaxation times of different compartments are not really known, and they might vary, depending on the type of lesion. In this study, we therefore decided not to correct for compartment-specific T2 relaxation times, as doing so would have introduced an additional degree of freedom for estimating g-ratio values.

Third, the estimates of the intracellular and isotropic signal fractions obtained from the NODDI processing might be influenced by compositional differences between lesions and healthy tissue (Lucchinetti et al., 1996). In (chronic) lesions, the number of astrocytes and other glial cells might possibly be increased (Frohman et al., 2006; Holley et al., 2003), which would influence the diffusion of water molecules, and thereby the signal fractions obtained from NODDI modeling. Depending on their permeability, glial cells could either contribute to the intracellular compartment, leading to an overestimation of the AVF or to the extracellular compartment (Zhang et al., 2012), leading to an underestimation of the AVF. Hence, the influence of such glial cells on water diffusion and resulting signal fractions is rather complex and requires further investigation.

Finally, assumptions about the general model for estimating AVFs (see Eq. (6)) could be erroneous in lesion tissue. This model assumes that the macromolecular pool is the same as the myelin pool (Mohammadi and Callaghan, 2021). Although this assumption seems to be approximately correct in healthy tissue, it might not hold true in lesions, where the content of non-myelin macromolecules could be elevated (Diaz-Sanchez et al., 2006). As a result, the volume fraction of non-myelin macromolecules (NMVF) might become non-negligible, and the general expression to calculate AVF (Eq. (6)) would extend to

(7) $$AVF=(1-MVF-NMVF)\cdot \left(1-v_{iso}\right)\cdot v_{ic}.$$

In this scenario, by neglecting the volume of non-myelin macro-molecules, both the AVF and the resulting g-ratio values would be overestimated in MS lesions when compared to NAWM. Alternatively, non-myelin macromolecules could be detected by the myelin-sensitive measures (such as MT-based measures or MTV) and contribute to the MVF leaving AVF rather unaffected, which would also lead to an underestimation of the g-ratio. In our data, however, the g-ratio values in lesions were lower than NAWM for three out of five myelin measures (MWF_NNLS_, ihMTR, and MTV) and higher for the remaining two measures (MWF_SPIJN_ and MTsat). In particular, the opposing behavior of MWF_NNLS_ vs. MWF_SPIJN_ and ihMTR vs. MTsat indicates that further influences certainly need to be considered. In this context, future studies need to explore the effect of structural and compositional changes due to pathology on the validity of the models and methods for myelin- and axonal-sensitive MRI, and finally g-ratio mapping. Until these issues are resolved, any g-ratio results on MS pathology need to be interpreted with caution.

### 4.7. General limitations of this study

The limitations of this study were the rather low number of participants and, in particular, the rather low EDSS scores of our MS patients. Moreover, parameter values were averaged across all lesion segmentations without considering different lesion types. As a result, this study does not allow for meaningful conclusions concerning MS pathologies, but it rather focuses on the methodological aspects of MRI-based g-ratio mapping and the influence of different myelin-sensitive measures. Another limitation of this study is the different spatial resolution of the acquired MRI measures, which influences registration of data sets and can lead to differential partial volume effects. We have attempted to address these issues by eroding lesion segmentations by one voxel. However, as resolutions ranged between 1 × 1 × 1 mm^3^ isotropic to 1 × 2 × 5 mm^3^, remaining partial volume effects could still have affected g-ratio evaluations. Furthermore, orientation dependent susceptibility effects in WM could have affected different myelin measures in slightly different ways. Although the nerve fiber orientation effects on T2 relaxation are weaker than the effects on T2* (Kaczmarz et al., 2020), GRASE-based MWF was found to vary by approximately 35% for different white matter fiber orientations and to generally decrease with increasing fiber angles (Birkl et al., 2021). While MT parameters such as the apparent transverse relaxation constant of the semisolid pool have revealed similar orientation dependence with peak values around 30° - 50° and decreasing values with higher fiber angles (Pampel et al., 2015), ihMTR values were found to be maximized for fibers perpendicular to the main magnetic field (Girard et al., 2017; Morris et al., 2022).

Regarding MTV-based g-ratio estimation, one limitation is the calibration of the underlying PD data. The PD value in whole-brain WM was assumed to be 69% for each healthy volunteer and MS patient. However, this assumption might have been wrong, especially in normal-appearing WM of MS patients. Additionally, one limitation of the g-ratio mapping model applied in this study is that the g-ratio was assumed to be constant within each voxel (Stikov et al., 2015), whereas in reality there is sub-voxel heterogeneity (Campbell et al., 2018), especially in pathological tissue like MS lesions. The low resolution of MRI-based g-ratio evaluations (compared to histology) does not allow revealing differences in the degree of myelinations between individual nerve fibers, and thus, cannot provide a full picture of the variety of different g-ratio values within lesions.

Importantly, there are still open questions regarding the most accurate tissue model for MWF-based g-ratio calculations and the most suitable calibration method, as well as corresponding calibration values, for MT-based g-ratio mapping (see Section 4.5). So far, mostly a single-point calibration has been used for MT-based g-ratio imaging (Ellerbrock and Mohammadi, 2018; Mohammadi et al., 2015; York et al., 2021), but two-point calibration has been suggested for improving g-ratio values (Mohammadi and Callaghan, 2021). However, two-point calibration requires a second reference value within another brain region, which could come at the expense of additional confounding factors since the g-ratio within different brain regions could vary between different subjects, especially in pathology. Besides, the assumed linear relationship between macromolecular and myelin content in MT-based or MTV-based g-ratio mapping could prove invalid, especially in disease (Campbell et al., 2018). However, while such differences in scaling would affect absolute values in the calculated g-ratio maps they will not change overall tendencies and differential distribution of the g-ratio values across tissue types. Future studies should combine several myelin-sensitive MRI measures with histology to evaluate the accuracy of each of the proposed models for MVF calculation and evaluate which of these methods is most accurate in both healthy and pathological tissue.

## 5. Conclusion

In this study, we found a high similarity of myelin-sensitive as well as derived g-ratio measures across healthy and normal-appearing WM. However, depending on the employed myelin measure and tissue model or calibration method used for g-ratio mapping, the g-ratio values varied greatly, especially within MS lesions. These results challenge the applicability of g-ratio MRI. For neuroscientific and clinical applications, it could be helpful to combine several complementary myelin-sensitive measures to obtain a broader picture of the condition of brain tissue and possible disease-related changes. In this context, MWF and MTsat could be a promising combination with MWF maps showing larger differences between several WM VOIs compared to other myelin measures and MTsat enabling a clear differentiation between lesion, perilesion, and NAWM at a high spatial resolution.

In general, when applying and comparing g-ratio values, it is important to use the same MRI methods and models for MVF and AVF mapping, or to consider methodological differences. Overall, this study highlights the need for evaluating the validity of methods developed on healthy data when they are applied to pathology. Future studies are needed which include both several different myelin-sensitive measures and gold standard histological measurements in order to disentangle processing-based influences from pathological alterations and evaluate the validity and accuracy of different g-ratio mapping methods in disease.

## Supplementary Material

1

## Figures and Tables

**Table 1:** Demographic and clinical details of study participants.

	Number	Gender	Average age [years] mean ± std. (range)	MS type	EDSS (range)	Disease duration [years] (range)
Healthy controls	14	11f / 3m	31.9 ± 7.9 (23 – 47)	–	–	–
MS patients	13	8f / 5m	34.3 ± 8.2 (24 – 46)	11 RRMS, 1 SPMS, 1 CIS	1.1 ± 1.3 (0 – 4.5)	8 ± 5 (1 – 15)

Abbreviations: MS: multiple sclerosis, EDSS: Expanded Disability Status Scale, RRMS: relapsing-remitting MS, SPMS: secondary progressive MS, CIS: clinically isolated syndrome.

**Table 2:** Comparison of average MVF and g-ratio values within healthy white matter (WM), normal-appearing white matter (NAWM), and multiple sclerosis lesions found in the current and in previous studies.

Publication	Cohort	Myelin measure	Axonal measure	MVF in healthy WM	g-ratio in healthy WM	MFV in NAWM	g-ratio in NAWM	MVF in lesion (range)	g-ratio in lesion (range)
This study	13 MS, 14 HC	MWF_NNLS_	NODDI	0.19	0.85	0.17	0.85	0.17 (0.07–0.26)	0.74 (0.65–0.90)
This study	13 MS, 14 HC	MWF_SPIJN_	NODDI	0.23	0.81	0.21	0.82	0.09 (0.02–0.23)	0.86 (0.72–0.96)
This study	13 MS, 14 HC	ihMTR	NODDI	0.35	0.72	0.33	0.72	0.26 (0.20–0.41)	0.64 (0.53–0.8)
This study	13 MS, 14 HC	MTsat	NODDI	0.39	0.69	0.38	0.68	0.18 (0.14–0.23)	0.72 (0.68–0.82)
This study	13 MS, 14 HC	MTV	NODDI	0.31	0.75	0.31	0.73	0.21 (0.18–0.26)	0.68 (0.60–0.75)
(Campbell et al., 2018)	1 MS, 5 HC	MTsat	NODDI	N/A	0.76	N/A	0.76	N/A	0.75 (0.72–0.8)
(Campbell et al., 2018)	1 MS, 5 HC	MTR	NODDI	N/A	0.76	N/A	0.76	N/A	0.65
(Hagiwara et al., 2017a)	20 MS	SyMRI	NODDI	N/A	N/A	0.31 ± 0.04	0.76 ± 0.05	0.06 ± 0.05	0.93 ± 0.06
(Maekawa et al., 2022)	31 MS	SyMRI	NODDI	N/A	N/A	~ 0.34	~ 0.7	~ 0.05 (0–0.22	~ 0.93 (0.8–1)
(Stikov et al., 2015)	1 MS, 1 HC	qMT / F	NODDI	N/A	0.7	N/A	0.75	N/A	0.8 [new lesions > 0.8]
(York et al., 2021)	73 MS	MTsat	NODDI	N/A	N/A	N/A	0.57	N/A	0.61 (0.54–0.68)
(Yu et al., 2019)	30 MS, 19 HC	MTV	ADM via SMT	0.27	0.66	0.25	0.67	0.17	0.74

References as well as measured MVF and g-ratio values are provided along with the study cohorts and methods used to determine the myelin and axonal volume fractions. NODDI: neurite orientation dispersion and density imaging, ADM: axon diameter mapping, SMT: Spherical Mean Technique, HC: healthy control, N/A: not available.

## Data Availability

In line with local ethics guidelines and participant privacy policies, sharing of acquired data will be considered upon reasonable request. Institutional policies would then require a formal data sharing agreement. For NNLS-based MWF calculation, the MATLAB scripts can be obtained from https://mriresearch.med.ubc.ca/news-projects/myelin-water-fraction/ and the Decaes toolbox provides a Julia-based equivalent for data processing (https://github.com/jondeuce/DECAES.jl). The processing script for ihMTR calculation can be made available upon request. The software for SPIJN-based MWF calculation requires a formal research agreement with Philips. A demo version is available via https://github.com/MNagtegaal/SPIJN. The latest version of the hMRI toolbox, which was used for calculation of MTsat and PD parameter maps, is available from www.hMRI.info. The latest version of the NODDI toolbox can be downloaded from https://www.nitrc.org/projects/noddi_toolbox. Sharing of any sequence modification applied here is limited by a nondisclosure agreement with the scanner manufacturer.

## Data Availability

The data that has been used is confidential.

## List of abbreviations

AVF: axonal volume fraction
DWI: diffusion-weighted imaging
FLAIR: fluid-attenuated inversion recovery
GM: gray matter
GRASE: gradient and spin echo
HC: healthy control
ihMT: inhomogeneous magnetization transfer
ihMTR: inhomogeneous magnetization transfer ratio
MPRAGE: magnetization prepared rapid gradient echo
MRI: magnetic resonance imaging
MS: multiple sclerosis
MT: magnetization transfer
MTsat: magnetization transfer saturation
MTV: macromolecular tissue volume
MVF: myelin volume fraction
MWF: myelin water fraction
MWI: myelin water imaging
NAWM: normal-appearing white matter
NMVF: volume fraction of non-myelin macromolecules
NNLS: non-negative least squares
NODDI: neurite orientation dispersion and density imaging
PD: proton density
SPIJN: sparsity promoting iterative joint non-negative least squares
VOI: volume of interest
WM: white matter
