# Supplementary material for: Comparing myelin-sensitive magnetic resonance imaging measures and resulting g-ratios in healthy and multiple sclerosis brains
Source: Neuroimage. Author manuscript; Available in PMC 2023 Feb 15. (PMC9931395; doi:10.1016/j.neuroimage.2022.119750)
Supplement: 1 [file NIHMS1860180-supplement-1.pdf]

Supplementary materials for:

Comparing myelin-sensitive magnetic resonance imaging measures and resulting g-ratios in healthy and multiple sclerosis brains

Supplementary methods

Image acquisition

Supplementary Table X1 provides a detailed overview of all acquired sequences and relevant imaging parameters. The parameter settings of both MT sequences were non-standard and required research software provided by the scanner manufacturer. The 3D gradient echo sequence for ihMT contained ten sinc-gauss-shaped pulses with pulse duration t_{ihMT} = 0.9 ms, MT saturation flip angle α_{ihMT} = 90°, (absolute) MT saturation off-resonance frequency f_{MT} = 7 kHz, at Δt_{ihMT} = 1.5 ms time intervals between successive MT pulses and required pulse sequence programming. The MTw sequence for multi-parameter mapping applied a single sinc-gauss-shaped MT pulse with MT pulse length t_{MT} = 12.8 ms, MT saturation flip angle α_{MT} = 540°, MT saturation off-resonance frequency f_{MT} = 2.2 kHz, water excitation pulse Δt_{ex} = 0.67 ms, and root mean square of the transmit B1+ pulse $B1_{rms}$ = 3.75 µT. For the diffusion data, an additional pair of non-diffusion-weighted volumes with b = 0 s/mm^2 with reverse phase encoding was acquired.

	**FLAIR**	**MPRAGE**	**MWI**	**ihMT**	**B1 map**	**T1w**	**PDw**	**MTw**	**DWI**
Image type	3D TSE	3D FFE (TFE)	3D SE (GRASE)	3D FFE (EPI)	3D FFE	3D FFE	3D FFE	3D FFE	MS SE (EPI)
Resolution [mm³]	(1.2)³	(1.0)³	1x2x5	(2.2)³	3.5x3.5 x5	(1.0)³	(1.0)³	(1.0)³	(2.0)³
FoV [mm³]	240x249 x168	256x240 x161	230x192 x100	211x211 x161	240x240 x175	240x240 x176	240x240 x176	240x240 x176	224x224 x140
# slices	140	161	40	73	70	176	176	176	70
# echoes	1	1	48	3	1	6	6	6	1
TE1 / ΔTE [ms]	276	3.7	8 / 8	3.5 / 5.7	2.3	2.4 / 2.4	2.4 / 2.4	2.4 / 2.4	97
TR [ms]	4800	8.1	1120	108	30 / 150	18	18	35	6426
Flip angle [°]	90	8	90	15	60	25	4	6	90
Water-fat shift [pix]	0.386	2.268	2.263	1.984	0.870	0.901	0.901	0.901	14.810
TSE/TFE factor	170	159	48	-	-	-	-	-	-
EPI factor	-	-	3	5	-	-	-	-	55
MB factor	-	-	-	-	-	-	-	-	2
Halfscan	no	no	no	no	no	Y: 0.6, Z:1	Y: 0.625, Z:1	Y: 0.6, Z:1	1
SENSE/CS	CS: 5	CS: 3	SEN: 2	SEN: 2.4	CS: 6	CS: 6	CS: 6	CS: 6	SEN: 2
Fold-over	AP	AP	RL	RL	AP	AP	AP	AP	AP
Fat shift dir.	F	F	L	L	F	F	F	F	P
Scan duration [min:s]	04:24	03:42	07:50	05:45	03:04	01:34	01:39	03:04	11:47

Supplementary Table X1: Overview of imaging parameters of all sequences in the study protocol. Abbreviations: FoV: field of view, TE: echo time, TR: repetition time, TSE: turbo spin echo, TFE: turbo field echo, FFE: fast field echo, EPI: echo-planar imaging, MB: multi band, CS: Compressed SENSE, SEN: SENSE, dir: direction, GRASE: gradient and spin echo, MS: multi-slice, SE: spin echo, AP: anterior-posterior, RL: right-left, F: foot, L: left, P: posterior. The fold-over direction is the primary phase-encoding direction.

Data processing

Myelin Water Imaging: Evaluation of spin echo trains for MWF assumes that the signal in an imaging voxel originates from spin ensembles with a range of T2 values, depending on their environment. We used two different approaches to determine MWF, "MWF_{NNLS}" and "MWF_{SPIJN}". MWF_{NNLS} was determined using a MATLAB implementation of a non-negative least squares (NNLS) algorithm (MacKay et al., 2006) provided by the UBC MRI Research Group (https://mriresearch.med.ubc.ca/news-projects/myelin-water-fraction/). Using NNLS, signal fractions of the T2 distributions (with 40 fixed logarithmically spaced T2 values ranging from 5 ms to 2 seconds) were determined and the MWF_{NNLS} was calculated as the sum of the signal fractions below a T2 cut-off of 40 ms. MWF_{SPIJN} was obtained using the "MWF analyzer" (version 1.3, 2020, Philips Research Hamburg), a dictionary-based approach using a combination of a non-negativity and a joint sparsity constraint (Nagtegaal et al., 2020). This method uses a pre-computed dictionary containing simulated signal evolutions to determine the T2 distributions from both magnitude and phase data. Utilized parameters were a SPIJN lambda of 40 and a T2 cut-off of 40 ms (same as for calculating the MWF_{NNLS}).

Inhomogeneous MT: Macromolecules saturated by an RF pulse can transfer energy to the free water pool, which is known as the MT effect. This macromolecular pool has a broad NMR spectral line due to the limited motion of the protons bound in lipid membranes (Girard et al., 2015). Dipolar coupling between protons on the long lipid chains can give rise to the so-called inhomogeneous magnetization transfer (ihMT) effect (Manning et al., 2017). The extent of this effect can be measured by applying MT pulses symmetrically around the resonance frequency. In our work, we calculated the ihMTR based on the four different MT-weighted images (Girard et al., 2015), two with single MT saturation pulses – applied at (either positive or negative) frequency offsets – and two with dual frequency offsets alternating between positive and negative (or negative and positive). To obtain the ihMTR, the two dual frequency offsets images are subtracted from the sum of the two single pulse MT images and the result is divided by the unsaturated free water image (Girard et al., 2015). This calculation was performed using a MATLAB-based script (provided by Guillaume Gilbert, Philips Healthcare Canada, 2017) incorporating the data from all three acquired echoes. The script used SPM12 for rigid-body registration of the different volumes and did not apply any low-pass filtering.

Multi-parameter mapping (PD, MTsat): Quantitative proton density maps were calculated using the hMRI toolbox (version v0.1.3-dev; https://github.com/tleutritz-cbs/hMRI-toolbox) and the Statistical Parametric Mapping (SPM) framework (SPM12, version v7771; https://www.fil.ion.ucl.ac.uk/spm/software/spm12/) based on 3D multi-echo gradient echo data sets with T1-, PD-, and MT-weighting and the B1 map. The data sets were reoriented via rigid-body transformation using the "Auto-reorient" function provided by the hMRI toolbox to correct for angulations of the head and to place the origin at the anterior commissure. Imperfect spoiling correction was performed based on the utilized phase increment of 150°. Default configuration parameters were used, except for a threshold of 10^8 for the PD map before bias field correction (which is scanner dependent).

Similar to ihMTR, magnetization transfer saturation (MTsat) also relies on the MT effect. It determines the signal decrease induced by a single MT saturation pulse in relation to an unsaturated free water image (Helms and Piringer, 2005). The MTsat maps were calculated according to (Helms et al., 2008), with the inclusion of the B1 transmit (B1+) field to correct for spatial deviations of the excitation flip angle. The remaining B1+ inhomogeneity in the images arising from the saturation pulses was corrected using a model-based approach (Rowley et al., 2021). T1-, PD-, and MT-weighted data extrapolated to an echo time of TE = 0 ms (obtained from the hMRI toolbox) and the B1 map were used to calculate maps of the apparent size of the bound pool ($M0B_{app}$), which can be used to characterize the relative change in MTsat at different RF powers (Rowley et al., 2021), and of the longitudinal relaxation rates (R1). Based

on the MT imaging parameters (as provided in the "Imaging acquisition" section), MTsat values were then simulated for a range of $B1_{rms}$, R1, and $M0B_{app}$ values from a single healthy volunteer. Finally, all MT-weighted data sets were corrected for B1+ inhomogeneities based on the simulated MTsat values and, for each data set individually, on the calculated R1 and measured B1 maps.

Neurite density estimation: Evaluation of DWI data for the intracellular and isotropic volume fractions v_{ic} and v_{iso}, was based on the NODDI model. According to this model, brain tissue is composed of three different compartments characterized by distinct properties of water diffusion, namely restricted diffusion for the intracellular compartment, hindered diffusion for the extracellular compartment, and isotropic diffusion for cerebrospinal fluid (CSF), thus resulting in diffusion signals with distinct properties. The intracellular compartment is modeled by cylinders of zero radius or sticks. The volume fraction of the intracellular compartment, v_{ic}, ranges between 0 and 1 and is interpreted as a neurite density index in WM. The orientation distribution of these sticks is modeled by a Watson distribution and yields an index of the orientation dispersion of neurites called orientation dispersion index (ODI), which characterizes the angular variation of neurite orientations in white matter and ranges from 0 to 1.

To perform these evaluations, we used the PreQual (Cai et al., 2021) software package, which combines both DWI and T1-weighted images to preprocess DWI data using MRtrix3 (Veraart et al., 2016), FSL (Jenkinson et al., 2012) version 6.0.1, and ANTs (Avants et al., 2009) version 2.0.1 software packages. The first step includes denoising using the "dwidenoise" function from MRtrix3. Then, a synthetic undistorted echo-planar image (EPI) is generated from T1-weighted and raw EPI data (b = 0) using deep learning (Schilling et al., 2020) and applied to correct susceptibility-induced distortions using FSL top-up (Andersson et al., 2003). As the third step, motion and eddy current-induced artefacts were corrected using FSL EDDY, including slice-to-volume motion correction (Andersson et al., 2017; Andersson and Sotiropoulos, 2016), outlier detection and replacement (Andersson et al., 2016) as well as b-vector rotation (Leemans and Jones, 2009). Following preprocessing, the intracellular and isotropic volume fractions, v_{ic} and v_{iso}, were generated using the NODDI model with the NODDI MATLAB toolbox (Zhang et al., 2012) version 1.04.

Calculation of MVF maps

MVF from MWF: For estimating the MR-visible volume ratios of the myelin and non-myelin compartments, κ_{my} and κ_{nm} in Eq. (2) of the manuscript, Jung and colleagues used both a geometric approach based on the estimated number of lamella of a myelinated fiber and a mass-density approach based on myelin mass and density values found in the literature (Jung et al., 2018). In addition to the method suggested by Jung and colleagues, we investigated two other methods of obtaining g-ratio parameter values from MWF. West and colleagues also based their g-ratio calculations on Eq. (2) but assumed $\kappa_{my} = 0.475 \frac{ml\, H_2O}{ml\ tissue}$ for the MR-visible volume ratio of myelin (West et al., 2018). Drakesmith and colleagues used a similar method and calculated MVF according to (Drakesmith et al., 2019)

$$MVF_{MWF} = \frac{MWF \cdot (1 + w)}{MWF \cdot w + 1},$$

where w is the lipid-water ratio in the myelin compartment, which was found to be $w = 0.72$ (Agrawal et al., 2009). However, Drakesmith and colleagues parameterized w in a slightly different way and assumed no dependence on the number of myelin sheaths (Drakesmith et al., 2019).

These various approaches yielded slightly higher g-ratio values (see Supplementary Figure S1), which agreed less with values found in the literature than the g-ratio values obtained using the method suggested by (Jung et al., 2018). We thus decided to use Eq. (2) of the manuscript and the MR-visible volume ratios suggested by Jung and colleagues for our study.

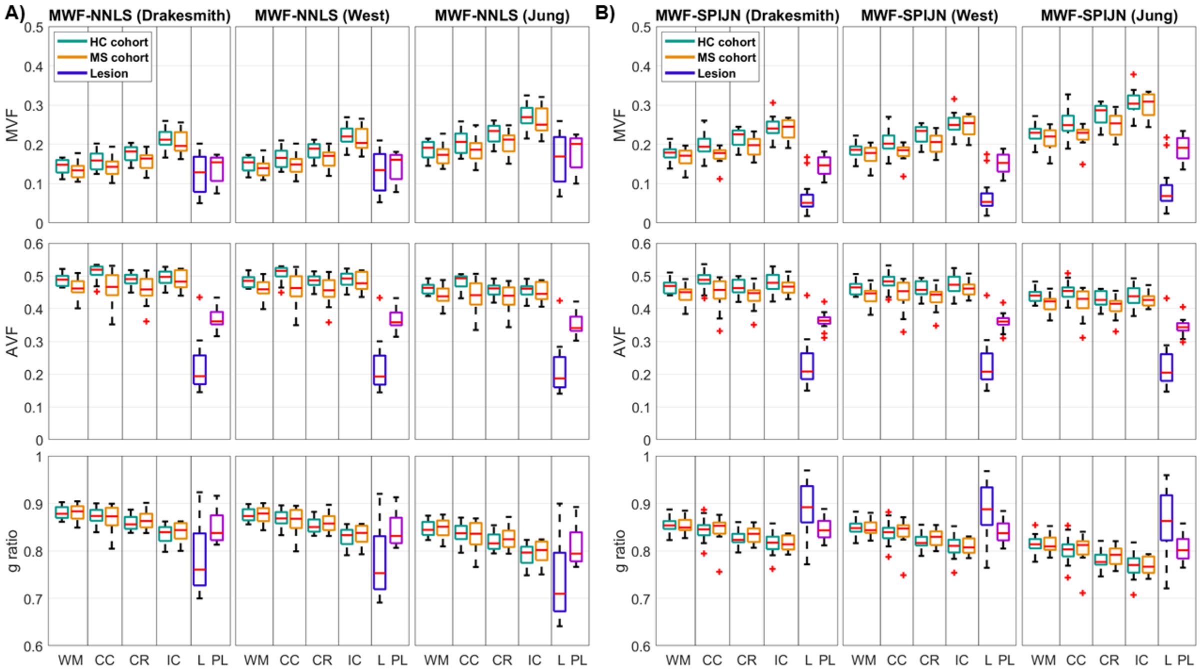

Supplementary Figure S1: Comparison of subject-mean MWF-based MVF, AVF, and g-ratio values calculated using three different approaches and input parameters. The myelin volume fraction (MVF) was calculated from the NNLS- (A) and SPIJN-derived (B) myelin water fraction values using the approaches suggested by Drakesmith et al. and West et al., and the geometric approach suggested by Jung et al. (columns). The MVF values obtained (top row) were used for calculation of AVF (middle row) and g-ratio (bottom row). All parameters were evaluated in healthy (HC cohort) or normal-appearing (MS cohort) white matter regions, in segmented MS lesions, and in a 2-voxel wide shell around MS lesions ("perilesion"). Abbreviations: WM: whole-brain WM, CC: corpus callosum, CR: corona radiata, IC: internal capsule, L: lesion, PL: perilesion.

<u>MVF from MT</u>: MT-based contrasts are not uniquely specific to myelin, but rather to the entire macromolecular compartment. Nevertheless, a high correlation with myelin content has been demonstrated, e.g., by (Schmierer et al., 2008). MTsat, the fractional decline of the tissue signal due to a simple MT pulse (Henkelman et al., 2001) as calculated according to (Helms et al., 2008), reduces dependencies on sequence parameters and T1, which improves the specificity of the MT contrast to the macromolecular pool (Helms et al., 2008) compared to the standard magnetization transfer ratio (MTR). Inhomogeneous MTR further increases the specificity to myelin (Varma et al., 2015) because it considers the dipolar nature of the myelin lipid's MT effect (Girard et al., 2015).

In this study, we used a simple scaling relation to obtain MVF from the MT-based myelin-sensitive measures (see Eq. (3) and Eq. (4) in the manuscript) in accordance with previous studies (Ellerbrock and Mohammadi, 2018; Emmenegger et al., 2021; Hara et al., 2020; Mohammadi et al., 2015; York et al., 2021). More generally, a linear relationship including a constant offset, c_{MT}, has been proposed for estimating MVF from MT-based measures (Campbell et al., 2018; Mohammadi and Callaghan, 2021)

$$MVF_{MT} = \alpha_{MT} \cdot MT + c_{MT} \ .$$

However, this constant offset has mostly been assumed to be close to zero and thus negligible (Ellerbrock and Mohammadi, 2018; Emmenegger et al., 2021; Hara et al., 2020; Mohammadi et al., 2015; York et al., 2021).

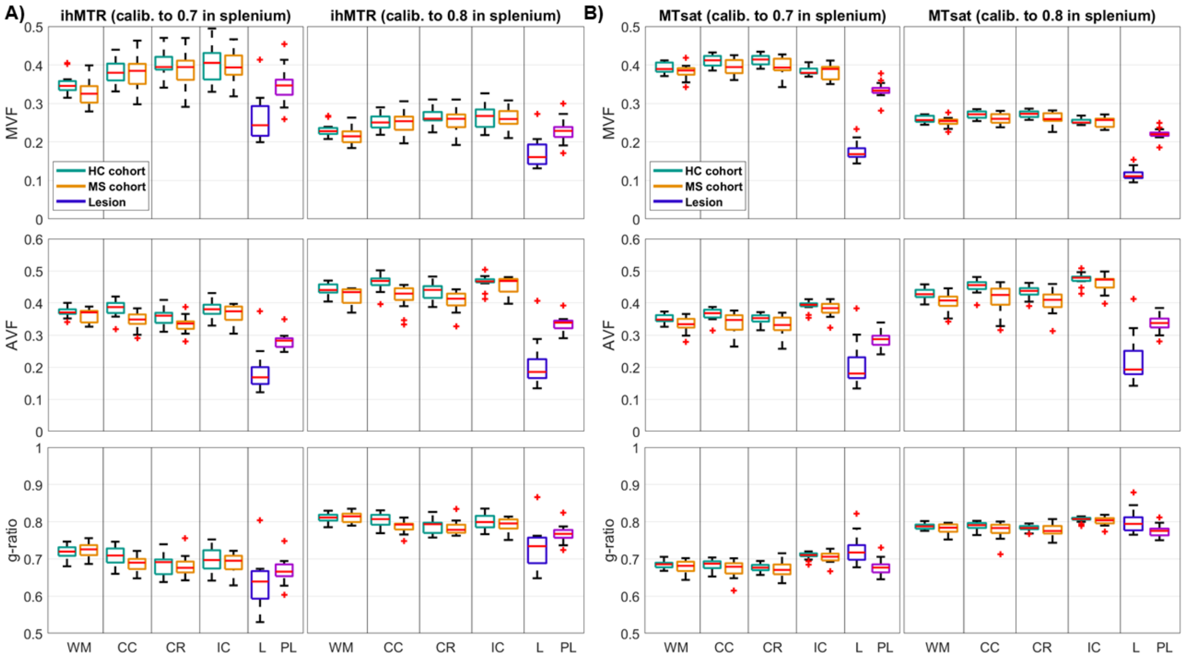

Supplementary Figure S2: Comparison of subject-mean MT-based MVF, AVF, and g-ratio values calculated using two different reference values for calibration. The myelin volume fraction (MVF) was calculated from the ihMTR- (A) and MTsat-derived (B) myelin measures scaled by calibrating the resulting g-ratio maps of the cohort of healthy volunteers to a value of either 0.7 or 0.8 within the splenium (columns). The obtained MVF values (top row) were used for calculation of AVF (middle row) and g-ratio (bottom row). All parameters were evaluated in healthy (HC cohort) or normal-appearing (MS cohort) white matter regions, in segmented MS lesions, and in a 2-voxel wide shell around MS lesions (“perilesion”). Abbreviations: WM: whole-brain WM, CC: corpus callosum, CR: corona radiata, IC: internal capsule, L: lesion, PL: perilesion.

<u>MVF from MTV</u>: MTV is estimated from proton density (Duval et al., 2017)

$$MTV = 1 - \frac{PD\ [\%]}{100\ \%} \ ,$$

implying contributions from macromolecules and lipids, from both myelin and non-myelin. Thus, similar to MT-based myelin measures, a linear calibration seems appropriate, i.e., $MVF = \alpha_{MTV} \cdot MTV$ (Ellerbrock and Mohammadi, 2018). However, many studies found a calibration constant close to one, which suggests that non-myelin macromolecules approximately compensate for myelin water and that MTV can be used without any scaling to estimate MVF (Berman et al., 2018; Duval et al., 2017; Yu et al., 2019). Since the g-ratio values from the latter approach agreed well with the values found in the literature, we decided not to apply any scaling, but to use MTV directly as a proxy for the MVF.

<u>VOI definition</u>

<u>Lesion segmentation</u>: For the lesion growth algorithm, default values were used including an initial threshold (kappa) of 0.3 and a Markov Random Field parameter of 1. Individual lesions were obtained using the MATLAB “bwconncomp" function, which was applied to find connected components within the lesion mask. The resulting individual lesions were eroded with a spherical structuring element of radius one voxel to reduce partial volume effects caused by the registration of data sets and by differences in spatial resolution. Furthermore, CSF segmentations dilated with a sphere-shaped structuring element of radius two voxels were subtracted from the lesion VOIs to remove partial volume influences from CSF.

Perilesion: First, the MATLAB "imdilate" function was applied to the lesion mask with a sphere-shaped structuring element of radius two voxels. The lesion voxels were then excluded from the perilesion mask obtained. Finally, this mask was intersected with the whole-brain WM VOI to obtain the perilesion tissue within normal-appearing white matter.

Whole-brain GM and WM: Lesion filling was applied to the MPRAGE data using the lesion segmentation tool (LST) toolbox to remove lesion voxels before segmentation into GM and WM tissue segments.

Atlas-based WM VOIs: The atlas-based WM VOIs were registered to the participants' MPRAGE data using the SPM12 "normalize" module via inverse deformation and nearest-neighbor interpolation. Atlas-based VOIs were then eroded with a sphere-shaped structuring element of one voxel and intersected with the whole-brain WM VOI.

Quantitative evaluations and image registration

The reference image for registration of the MWF maps to the MPRAGE was the brain-masked first echo of the T2w 3D GRASE. The ihMTR processing provided a gradient echo-based reference image and the hMRI toolbox an R1 map, which were used for registration of the ihMTR map and the hMRI toolbox-based MTsat and MTV maps, respectively. Finally, the intracellular and isotropic volume fraction maps were registered to the MPRAGE data using the SPM "coregister" function and the b0 diffusion image as a reference.

All quantitative evaluations were performed within a subject-specific, common brain mask calculated as the intersection of brain masks obtained from both the 3D GRASE data and the b0 map from the diffusion-weighted data.

Two-sample *t*-tests were performed using the MATLAB "ttest2" function and Pearson correlation coefficients via the MATLAB "corrcoef" function.

Supplementary results

***p*-value**	**HC - MS**	**HC - MS**	**HC - MS**	**HC - MS**	**HC - MS**	**HC - MS**	**HC - MS**	**MS**	**MS**	**MS**
	**WM**	**CC**	**CR**	**IC**	**SPL**	**EC**	**CIN**	**L - PL**	**L - WM**	**PL - WM**
**MWF_{NNLS}**	0.192	0.065	**0.027**	0.391	**0.025**	0.574	0.357	0.623	0.641	0.911
**MWF_{SPIJN}**	0.202	**0.029**	**0.027**	0.663	**0.034**	0.537	0.247	**<0.001**	**<0.001**	**0.030**
**ihMTR**	**0.047**	0.827	0.333	0.897	0.608	0.384	0.661	**0.001**	**0.002**	0.208
**MTsat**	0.153	**0.041**	0.050	0.501	**0.006**	0.285	0.885	**<0.001**	**<0.001**	**<0.001**
**MTV**	0.613	0.822	0.300	0.087	0.911	0.626	**0.003**	**<0.001**	**<0.001**	**<0.001**

Supplementary Table X2: *p*-values of two-sample *t*-tests of subject-mean myelin-sensitive measures within several volumes of interest (VOIs) or between VOIs. The two-sample *t*-tests were either performed on data of healthy volunteers (HC) and MS patients within the same VOI (first seven columns) or using data of MS patients from different VOIs (last three columns). *p*-values < 0.05 are highlighted in bold. WM: whole-brain WM, CC: corpus callosum, CR: corona radiata, IC: internal capsule, SPL: splenium, EC: external capsule, CIN: cingulum, L: lesion, PL: perilesion (2 voxels).

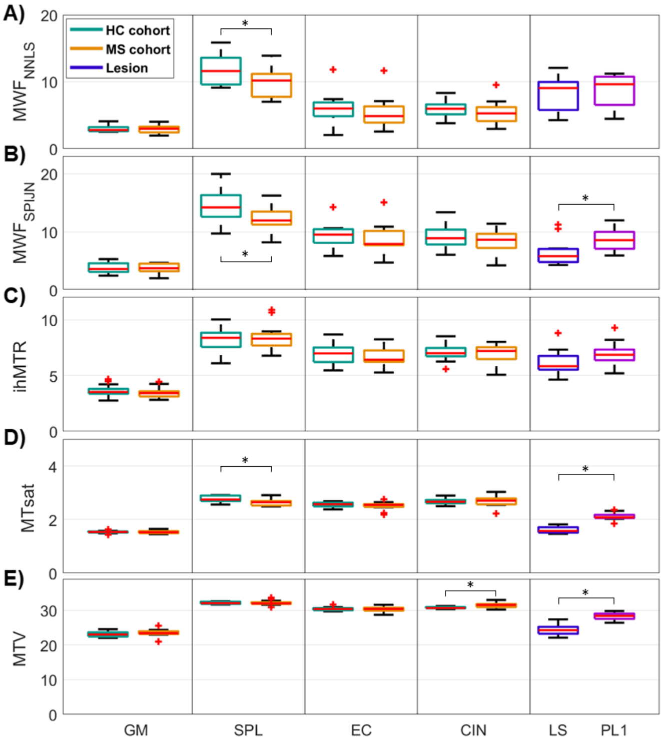

Supplementary Figure S3: Boxplots of subject-mean myelin-sensitive measure values within several additional VOIs. The values of MWF_{NNLS}, MWF_{SPIJN}, ihMTR, MTsat, and MTV were evaluated for healthy (HC cohort, green) and normal-appearing (MS cohort, orange) whole-brain GM segmentations, several atlas-based WM VOIs, in a 1-voxel wide shell within segmented MS lesions ("lesion shell", purple-blue), and in a 1 voxel wide shell around MS lesions ("perilesion", pink). In all five panels, the boxplots represent distributions across subjects. Significant differences between HC and MS cohorts within the same VOI or between MS WM, lesion, and perilesion are indicated by asterisks.
Abbreviations: GM: whole-brain GM, SPL: splenium, EC: external capsule, CIN: cingulum LS: lesion shell, PL1: perilesion (1-voxel).

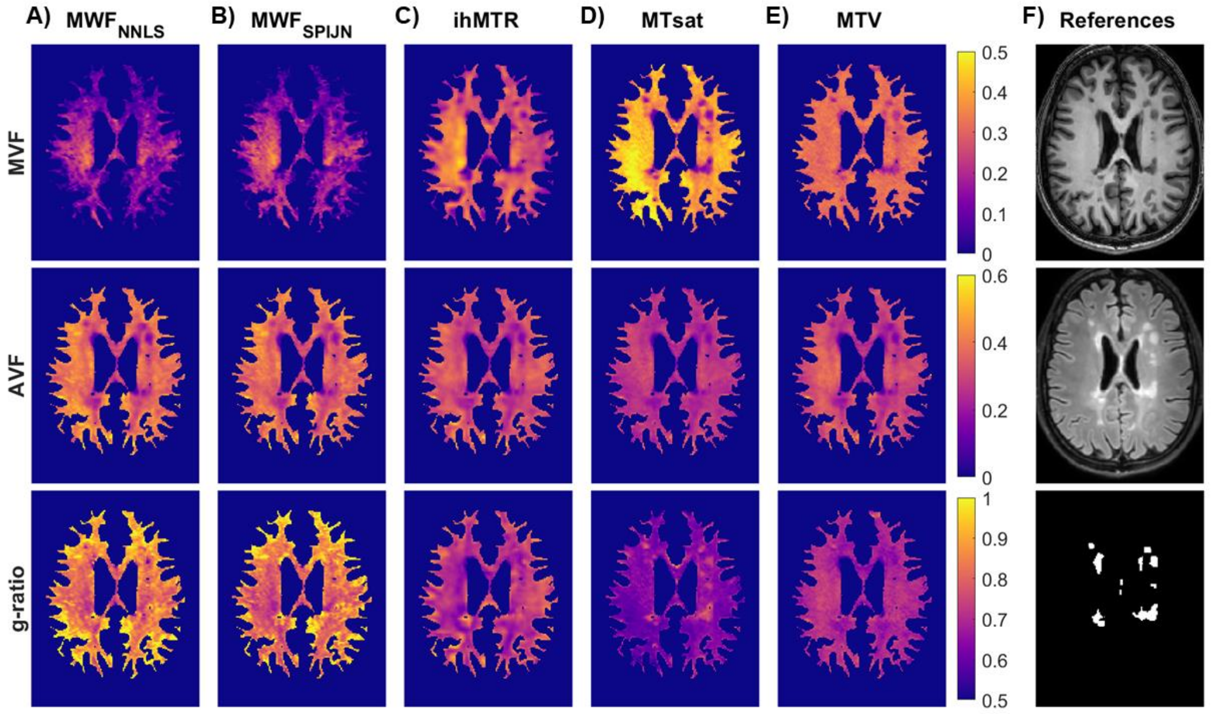

Supplementary Figure S4: Additional representative slice of myelin volume fraction (MVF), axonal volume fraction (AVF), and g-ratio parameter maps from an MS patient calculated based on different myelin-sensitive measures. Myelin-sensitive measures include MWI using two different fitting techniques for the myelin water fraction, MWF_{NNLS} (A) and MWF_{SPIJN} (B), as well as ihMTR (C), MTsat (D), and MTV (E). As reference, the MPRAGE (top), FLAIR (middle), and lesion mask (bottom) are shown in the last column (F).

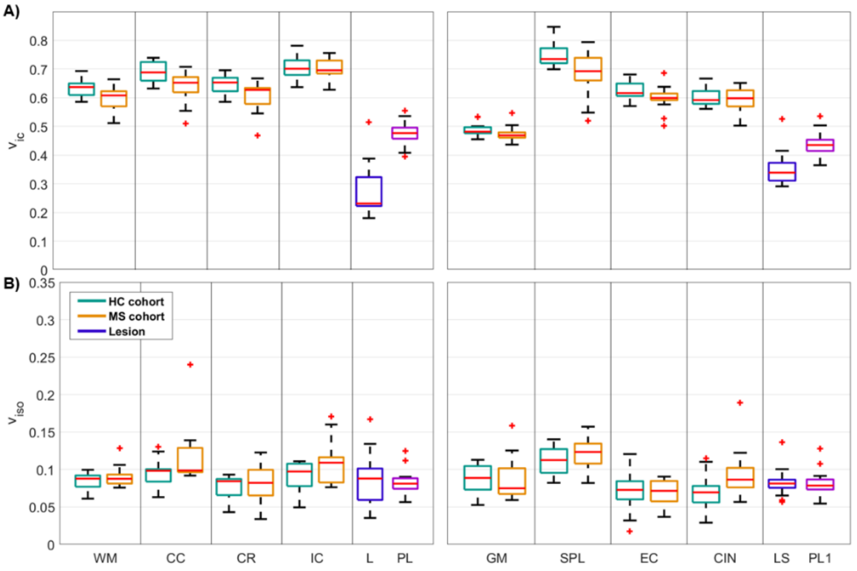

Supplementary Figure S5: Quantitative comparison of subject-mean intracellular, v_{ic}, and isotropic volume fractions, v_{iso}, in several brain regions. The volume fractions v_{ic} (A) and v_{iso} (B) were generated using NODDI and evaluated in healthy (HC cohort) or normal-appearing (MS cohort) white matter regions, in segmented MS lesions and in a 1 or 2-voxel wide shell around MS lesions ("perilesion"). Abbreviations: WM: whole-brain WM, CC: corpus callosum, CR: corona radiata, IC: internal capsule, L: lesion, PL: perilesion (2-voxel), GM: whole-brain GM, SPL: splenium, EC: external capsule, CIN: cingulum, LS: lesion shell, PL1: perilesion (1-voxel).

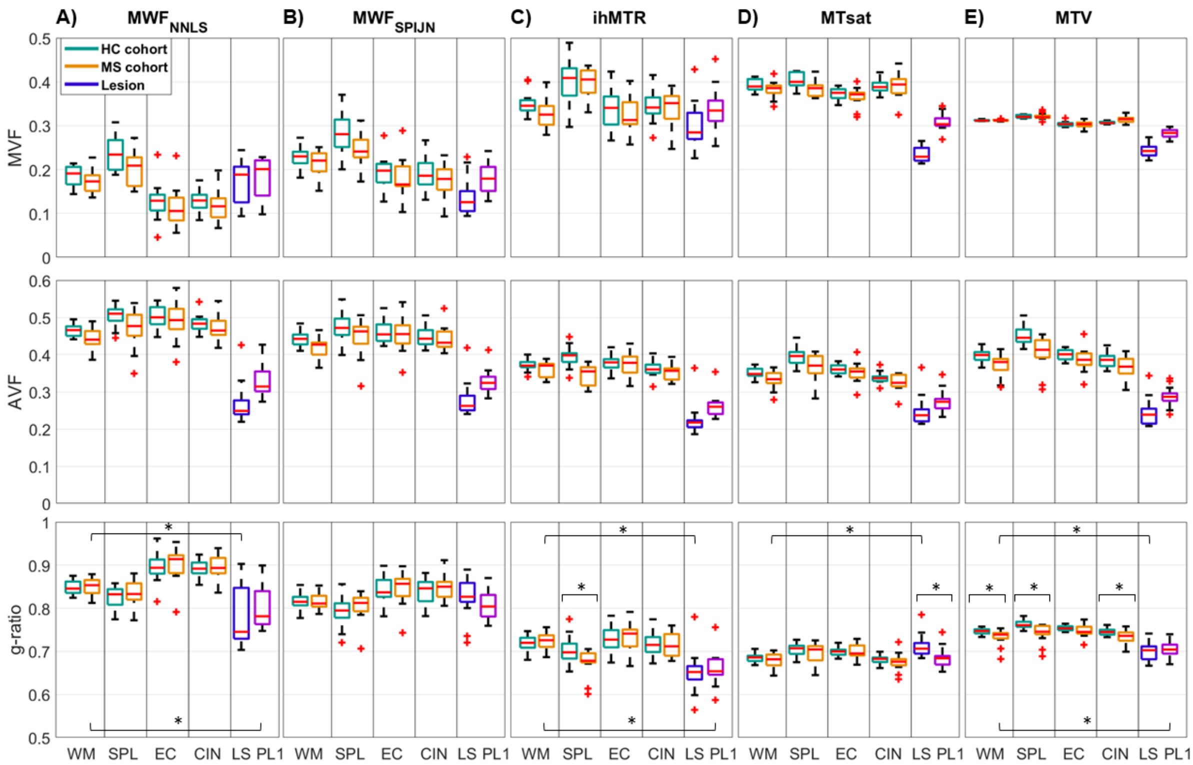

Supplementary Figure S6: Quantitative comparison of subject-mean MVF, AVF, and g-ratio values in several additional brain regions. The volume fractions were calculated for each of the five myelin-sensitive measures (columns) and evaluated in healthy (HC cohort) or normal-appearing (MS cohort) white matter regions, in the 1-voxel wide shell within segmented MS lesions ("lesion shell"), and in a 1-voxel wide shell around MS lesions ("perilesion"). For each of the five g-ratio measures, statistically significant differences between healthy WM and NAWM or between whole-brain NAWM and (peri-) lesion tissue are indicated by an asterisk. Abbreviations: WM: whole-brain WM, SPL: splenium, EC: external capsule, CIN: cingulum, LS: lesion shell, PL1: perilesion (1-voxel).

p-value	HC - MS	HC - MS	HC - MS	HC - MS	HC - MS	HC - MS	HC - MS	MS	MS	MS
	**WM**	**CC**	**CR**	**IC**	**SPL**	**EC**	**CIN**	**L - PL**	**L - WM**	**PL - WM**
**MWF_{NNLS}**	0.855	0.909	0.36	0.646	0.352	0.736	0.506	**0.013**	**<0.001**	**0.018**
**MWF_{SPIJN}**	0.912	0.676	0.288	0.983	0.46	0.722	0.368	**0.024**	0.060	0.256
**ihMTR**	0.491	**0.040**	0.721	0.629	**0.028**	0.722	0.868	0.154	**<0.001**	**<0.001**
**MTsat**	0.188	0.143	0.485	0.449	0.379	0.793	0.326	**0.001**	**0.001**	0.919
**MTV**	**0.015**	**0.019**	**0.016**	**0.029**	**0.004**	0.164	**0.018**	**0.040**	**0.001**	**0.003**

Supplementary Table X3: *p*-values of two-sample *t*-tests of subject-mean g-ratio within several VOIs or between VOIs. The two-sample *t*-tests were performed either using data of healthy volunteers (HC) and MS patients within the same VOI (first seven columns) or using data of MS patients from different VOIs (last three columns). *p*-values < 0.05 are highlighted in bold. WM: whole-brain WM, CC: corpus callosum, CR: corona radiata, IC: internal capsule, SPL: splenium, EC: external capsule, CIN: cingulum, L: lesion, PL: perilesion (2 voxels).

**Publication**	**Cohort**	**v_{ic} (WM/NAWM)**	**v_{ic} (lesion)**	**b-values [s/mm^2]**	**TE [ms]**	**TR [ms]**
**This study**	**13 MS, 14 HC**	**~ 0.6 - 0.62**	**~ 0.28**	**711, 2000**	**97**	**6445**
(Hagiwara et al., 2019)	24 MS, 24 HC	**~ 0.61**	**~ 0.34**	1000 & 2000	88.2	5000
(Andersen et al., 2018)	30 MS, 17HC	**~ 0-.62**	**~ 0.32**			
(Mustafi et al., 2019)	6 MS	0.7	0.45	250, 1000, 2250, 4000, 6250	114.24	3590
(Granberg et al., 2017)	24 MS, 26 HC	0.622 / 0.638	0.478	1000 & 5000	57	8800
(Kato et al., 2022)	30 MS, 20 HC	0.57 - 0.59	0.43 - 0.54	1000 & 2000	88.2	5000
(Johnson et al., 2021)	63 MS, 28 HC	~ 0.56	~ 0.53	300, 711, 2000	82	14 000

Supplementary Table X4: v_{ic} values within healthy white matter (WM) or normal-appearing white matter (NAWM) and multiple sclerosis lesions found in previous studies. Reference v_{ic} values are provided along with the study cohort, the b-values used, the echo time (TE), and the repetition time (TR). MS: multiple sclerosis patients, HC: healthy controls.

References

Agrawal, D., Hawk, R., Avila, R.L., Inouye, H., Kirschner, D.A., 2009. Internodal myelination during development quantitated using X-ray diffraction. J Struct Biol 168, 521-526. https://doi.org/10.1016/j.jsb.2009.06.019.

Andersen, K.W., Lasic, S., Lundell, H., Nilsson, M., Topgaard, D., Szczepankiewicz, F., Hanson, L.G., Siebner, H., Blinkenberg, M., Dyrby, T., 2018. Multi-dimensional microstructural imaging offers novel in vivo insights into brain pathology: an application to multiple sclerosis. Proc Intl Soc Mag Reson Med. https://core.ac.uk/download/pdf/158596035.pdf.

Andersson, J.L., Graham, M.S., Drobnjak, I., Zhang, H., Filippini, N., Bastiani, M., 2017. Towards a comprehensive framework for movement and distortion correction of diffusion MR images: Within volume movement. Neuroimage 152, 450-466. https://doi.org/10.1016/j.neuroimage.2017.02.085

Andersson, J.L., Graham, M.S., Zsoldos, E., Sotiropoulos, S.N., 2016. Incorporating outlier detection and replacement into a non-parametric framework for movement and distortion correction of diffusion MR images. Neuroimage 141, 556-572. https://doi.org/10.1016/j.neuroimage.2016.06.058

Andersson, J.L., Skare, S., Ashburner, J., 2003. How to correct susceptibility distortions in spin-echo echo-planar images: application to diffusion tensor imaging. Neuroimage 20, 870-888. https://doi.org/10.1016/S1053-8119(03)00336-7

Andersson, J.L., Sotiropoulos, S.N., 2016. An integrated approach to correction for off-resonance effects and subject movement in diffusion MR imaging. Neuroimage 125, 1063-1078. https://doi.org/10.1016/j.neuroimage.2015.10.019

Avants, B.B., Tustison, N., Song, G., 2009. Advanced normalization tools (ANTS). The Insight Journal 2, 1-35. https://doi.org/10.54294/uvnhin.

Berman, S., West, K.L., Does, M.D., Yeatman, J.D., Mezer, A.A., 2018. Evaluating g-ratio weighted changes in the corpus callosum as a function of age and sex. Neuroimage 182, 304-313. https://doi.org/10.1016/j.neuroimage.2017.06.076.

Cai, L.Y., Yang, Q., Hansen, C.B., Nath, V., Ramadass, K., Johnson, G.W., Conrad, B.N., Boyd, B.D., Begnoche, J.P., Beason-Held, L.L., 2021. PreQual: An automated pipeline for integrated preprocessing and quality assurance of diffusion weighted MRI images. Magn Reson Med 86, 456-470. https://doi.org/10.1002/mrm.28678

Campbell, J.S., Leppert, I.R., Narayanan, S., Boudreau, M., Duval, T., Cohen-Adad, J., Pike, G.B., Stikov, N., 2018. Promise and pitfalls of g-ratio estimation with MRI. Neuroimage 182, 80-96. https://doi.org/10.1016/j.neuroimage.2017.08.038.

Drakesmith, M., Harms, R., Rudrapatna, S.U., Parker, G.D., Evans, C.J., Jones, D.K., 2019. Estimating axon conduction velocity in vivo from microstructural MRI. Neuroimage 203, 116186. https://doi.org/10.1016/j.neuroimage.2019.116186.

Duval, T., Lévy, S., Stikov, N., Campbell, J., Mezer, A., Witzel, T., Keil, B., Smith, V., Wald, L.L., Klawiter, E., 2017. g-Ratio weighted imaging of the human spinal cord in vivo. Neuroimage 145, 11-23. https://doi.org/10.1016/j.neuroimage.2016.09.018.

Ellerbrock, I., Mohammadi, S., 2018. Four in vivo g-ratio-weighted imaging methods: Comparability and repeatability at the group level. Hum Brain Mapp 39, 24-41. https://doi.org/10.1002/hbm.23858.

Emmenegger, T.M., David, G., Ashtarayeh, M., Fritz, F.J., Ellerbrock, I., Helms, G., Balteau, E., Freund, P., Mohammadi, S., 2021. The Influence of Radio-Frequency Transmit Field Inhomogeneities on the Accuracy of G-ratio Weighted Imaging. Front Neurosci 15, 770. https://doi.org/10.3389/fnins.2021.674719.

Girard, O.M., Prevost, V.H., Varma, G., Cozzone, P.J., Alsop, D.C., Duhamel, G., 2015. Magnetization transfer from inhomogeneously broadened lines (ihMT): experimental optimization of saturation parameters for human brain imaging at 1.5 Tesla. Magn Reson Med 73, 2111-2121. https://doi.org/10.1002/mrm.25330.

Granberg, T., Fan, Q., Treaba, C.A., Ouellette, R., Herranz, E., Mangeat, G., Louapre, C., Cohen-Adad, J., Klawiter, E.C., Sloane, J.A., 2017. In vivo characterization of cortical and white matter neuroaxonal pathology in early multiple sclerosis. Brain 140, 2912-2926. https://doi.org/10.1093/brain/awx247.

Hagiwara, A., Kamagata, K., Shimoji, K., Yokoyama, K., Andica, C., Hori, M., Fujita, S., Maekawa, T., Irie, R., Akashi, T., 2019. White matter abnormalities in multiple sclerosis evaluated by quantitative synthetic MRI, diffusion tensor imaging, and neurite orientation dispersion and density imaging. AJNR Am J Neuroradiol 40, 1642-1648. https://doi.org/10.3174/ajnr.A6209

Hara, S., Hori, M., Hagiwara, A., Tsurushima, Y., Tanaka, Y., Maehara, T., Aoki, S., Nariai, T., 2020. Myelin and Axonal Damage in Normal-Appearing White Matter in Patients with Moyamoya Disease. AJNR Am J Neuroradiol 41, 1618-1624. https://doi.org/10.3174/ajnr.a6708

Helms, G., Dathe, H., Kallenberg, K., Dechent, P., 2008. High-resolution maps of magnetization transfer with inherent correction for RF inhomogeneity and T1 relaxation obtained from 3D FLASH MRI. Magn Reson Med 60, 1396-1407. https://doi.org/10.1002/mrm.21732.

Helms, G., Piringer, A., 2005. Simultaneous measurement of saturation and relaxation in human brain by repetitive magnetization transfer pulses. NMR Biomed 18, 44-50. https://doi.org/10.1002/nbm.920.

Henkelman, R., Stanisz, G., Graham, S., 2001. Magnetization transfer in MRI: a review. NMR Biomed 14, 57-64. https://doi.org/10.1002/nbm.683.

Jenkinson, M., Beckmann, C.F., Behrens, T.E., Woolrich, M.W., Smith, S.M., 2012. Fsl. Neuroimage 62, 782-790. https://doi.org/10.1016/j.neuroimage.2011.09.015.

Johnson, D., Ricciardi, A., Brownlee, W., Kanber, B., Prados, F., Collorone, S., Kaden, E., Toosy, A., Alexander, D.C., Gandini Wheeler-Kingshott, C.A., 2021. Comparison of Neurite Orientation Dispersion and Density Imaging and Two-Compartment Spherical Mean Technique Parameter Maps in Multiple Sclerosis. Frontiers in neurology 12, 944. https://doi.org/10.3389/fneur.2021.662855.

Jung, W., Lee, J., Shin, H.-G., Nam, Y., Zhang, H., Oh, S.-H., Lee, J., 2018. Whole brain g-ratio mapping using myelin water imaging (MWI) and neurite orientation dispersion and density imaging (NODDI). Neuroimage 182, 379-388. https://doi.org/10.1016/j.neuroimage.2017.09.053.

Kato, S., Hagiwara, A., Yokoyama, K., Andica, C., Tomizawa, Y., Hoshino, Y., Uchida, W., Nishimura, Y., Fujita, S., Kamagata, K., 2022. Microstructural white matter abnormalities in multiple sclerosis and neuromyelitis optica spectrum disorders: Evaluation by advanced diffusion imaging. J Neurol Sci 436, 120205. https://doi.org/10.1016/j.jns.2022.120205.

Leemans, A., Jones, D.K., 2009. The B-matrix must be rotated when correcting for subject motion in DTI data. Magn Reson Med 61, 1336-1349. https://doi.org/10.1002/mrm.21890.

MacKay, A., Laule, C., Vavasour, I., Bjarnason, T., Kolind, S., Mädler, B., 2006. Insights into brain microstructure from the T2 distribution. Magn Reson Imaging 24, 515-525. https://doi.org/10.1016/j.mri.2005.12.037.

Manning, A.P., Chang, K.L., MacKay, A.L., Michal, C.A., 2017. The physical mechanism of “inhomogeneous” magnetization transfer MRI. J Magn Reson 274, 125-136. https://doi.org/10.1016/j.jmr.2016.11.013.

Mohammadi, S., Callaghan, M.F., 2021. Towards in vivo g-ratio mapping using MRI: Unifying myelin and diffusion imaging. J Neurosci Methods 348, 108990. https://doi.org/10.1016/j.jneumeth.2020.108990.

Mohammadi, S., Carey, D., Dick, F., Diedrichsen, J., Sereno, M.I., Reisert, M., Callaghan, M.F., Weiskopf, N., 2015. Whole-brain in-vivo measurements of the axonal g-ratio in a group of 37 healthy volunteers. Front Neurosci 9, 441. https://doi.org/10.3389/fnins.2015.00441.

Mustafi, S.M., Harezlak, J., Kodiweera, C., Randolph, J.S., Ford, J.C., Wishart, H.A., Wu, Y.-C., 2019. Detecting white matter alterations in multiple sclerosis using advanced diffusion magnetic resonance imaging. Neural Regen Res 14, 114. https://doi.org/10.4103/1673-5374.243716.

Nagtegaal, M., Koken, P., Amthor, T., de Bresser, J., Mädler, B., Vos, F., Doneva, M., 2020. Myelin water imaging from multi-echo T2 MR relaxometry data using a joint sparsity constraint. Neuroimage 219, 117014. https://doi.org/10.1016/j.neuroimage.2020.117014.

Rowley, C.D., Campbell, J.S., Wu, Z., Leppert, I.R., Rudko, D.A., Pike, G.B., Tardif, C.L., 2021. A model-based framework for correcting inhomogeneity effects in magnetization transfer saturation and inhomogeneous magnetization transfer saturation maps. Magn Reson Med 86, 2192-2207. https://doi.org/10.1002/mrm.28831.

Schilling, K.G., Blaber, J., Hansen, C., Cai, L., Rogers, B., Anderson, A.W., Smith, S., Kanakaraj, P., Rex, T., Resnick, S.M., 2020. Distortion correction of diffusion weighted MRI without reverse phase-encoding scans or field-maps. PloS one 15, e0236418. https://doi.org/10.1371/journal.pone.0236418.

Schmierer, K., Wheeler-Kingshott, C.A., Tozer, D.J., Boulby, P.A., Parkes, H.G., Yousry, T.A., Scaravilli, F., Barker, G.J., Tofts, P.S., Miller, D.H., 2008. Quantitative magnetic resonance of postmortem multiple sclerosis brain before and after fixation. Magn Reson Med 59, 268-277. https://doi.org/10.1002/mrm.21487.

Varma, G., Duhamel, G., de Bazelaire, C., Alsop, D.C., 2015. Magnetization transfer from inhomogeneously broadened lines: a potential marker for myelin. Magn Reson Med 73, 614-622. https://doi.org/10.1002/mrm.25174.

Veraart, J., Novikov, D.S., Christiaens, D., Ades-Aron, B., Sijbers, J., Fieremans, E., 2016. Denoising of diffusion MRI using random matrix theory. Neuroimage 142, 394-406. https://doi.org/10.1016/j.neuroimage.2016.08.016

West, K.L., Kelm, N.D., Carson, R.P., Gochberg, D.F., Ess, K.C., Does, M.D., 2018. Myelin volume fraction imaging with MRI. Neuroimage 182, 511-521. https://doi.org/10.1016/j.neuroimage.2016.12.067.

York, E.N., Martin, S.-J., Meijboom, R., Thrippleton, M.J., Bastin, M.E., Carter, E., Overell, J., Connick, P., Chandran, S., Waldman, A.D., 2021. MRI-derived g-ratio and lesion severity in newly diagnosed multiple sclerosis. Brain Commun 3, fcab249. https://doi.org/10.1093/braincomms/fcab249.

Yu, F., Fan, Q., Tian, Q., Ngamsombat, C., Machado, N., Bireley, J.D., Russo, A.W., Nummenmaa, A., Witzel, T., Wald, L.L., 2019. Imaging G-ratio in multiple sclerosis using high-gradient diffusion MRI and macromolecular tissue volume. AJNR Am J Neuroradiol 40, 1871-1877. www.doi.org/10.3174/ajnr.A6283.

Zhang, H., Schneider, T., Wheeler-Kingshott, C.A., Alexander, D.C., 2012. NODDI: practical in vivo neurite orientation dispersion and density imaging of the human brain. Neuroimage 61, 1000-1016. https://doi.org/10.1016/j.neuroimage.2012.03.072.
